# Efficacy of sodium glucose cotransporter 2 inhibitors on hepatic fibrosis and steatosis in non-alcoholic fatty liver disease: an updated systematic review and meta-analysis

**DOI:** 10.1038/s41598-024-52603-5

**Published:** 2024-01-24

**Authors:** Albert Macaire C. Ong Lopez, Janine Audrei T. Pajimna

**Affiliations:** 1 Department of Medicine, Section of Endocrinology, Diabetes and Metabolism, St. Luke’s Medical Center- Quezon City, 279 E Rodriguez Sr. Ave, 1112 Quezon City, Metro Manila Philippines; 2 Department of Medicine, St. Luke’s Medical Center-Quezon City, 279 E Rodriguez Sr. Ave, 1112 Quezon City, Metro Manila Philippines

**Keywords:** Endocrine system and metabolic diseases, Diabetes, Non-alcoholic fatty liver disease

## Abstract

Non-alcoholic fatty liver disease (NAFLD) is a substantial contributor to liver-related morbidity worldwide, and yet, there are no standard, universal pharmacologic therapies approved for this indication. The aim of this systematic review and meta-analysis is to evaluate the effectiveness of SGLT-2 inhibitors in improving hepatic steatosis and hepatic fibrosis in patients with NAFLD. An extensive electronic database search was done to identify studies published from inception until December 2023, without any language restrictions. All randomized controlled trials (RCT) that evaluated the use of SGLT-2 inhibitors for patients with NAFLD, regardless of diabetes mellitus status, were included. The Cochrane Risk of Bias 2.0 tool was used to assess the risk of bias of each study included. Evidence from all studies were synthesized as mean differences for continuous data, and as risk ratio for dichotomous outcomes. An inverse variance or Mantel–Haenszel test was used in conjunction with a random-effects meta-analysis model, where necessary. 18 eligible RCTs involving 1330 participants were analyzed, all of which had risk of bias ranging from low to some concerns. Significant difference in means was observed for controlled attenuation parameter (6 trials, n = 372; MD: − 10.59 dB/m, 95% CI [− 18.25, − 2.92], *p* = 0.007, I^2^ = 0%); L/S ratio (3 trials, n = 163; MD: 0.11, 95% CI [0.01, 0.21], *p* = 0.04, I^2^ = 78%); LSM (7 trials, n = 447; MD: − 0.67 kPa, 95% CI [− 1.19, − 0.16], p = 0.010, I^2^ = 69%); MRI-PDFF (5 trials, n = 330; MD: − 2.61%, 95% CI [− 5.05, − 0.17], *p* = 0.04, I^2^ = 78%), and FIB-4 index (10 trials, n = 648; MD: − 0.12, 95% CI [− 0.21, − 0.04], *p* = 0.005, I^2^ = 16%) after SGLT-2 inhibitor treatment as compared to controls. In conclusion, the use of SGLT-2 inhibitors may lead to slight improvement of hepatic steatosis and/or fibrosis as compared to controls in patients with NAFLD and Type 2 diabetes mellitus based on imaging and histopathology biomarkers with low to moderate certainty of evidence.

## Introduction

Non-alcoholic fatty liver disease (NAFLD) is a major cause of liver-related morbidity globally, with prevalence rates as high as 30%^1^, and steadily increasing number of cases, from 391.2 million in 1990 to 882.1 million in 2017^2^.

It appears to be associated with one or more components of the metabolic syndrome, which include hypertension, dyslipidemia, obesity, and Type 2 diabetes mellitus and insulin resistance^1,3^. Although its pathogenesis has not yet been completely established, growing evidence shows that insulin resistance and dysregulation in lipid metabolism play huge roles in the development of hepatic steatosis. High fat diet, insulin resistance, obesity, dysregulated peripheral lipolysis, and other potential risk factors lead to increased entry of free fatty acids into the liver, which places hepatocytes under a ‘lipotoxic’ condition^4^. The accumulation of triacylglycerol in the hepatocyte cytoplasm presents histologically as steatosis. With the constant, repeated micro-hepatic injury, endoplasmic reticulum stress and mitochondrial dysfunction now arise. This then leads to lobular inflammation, cellular apoptosis, and hepatic fibrosis over time^4^.

If neglected, this treatable condition can lead to various severe consequences, such as advanced cirrhosis, hepatocellular cancer, and potentially cardiovascular morbidity^5^ and mortality^6,7^. Considering the prognostic consequences of NAFLD, effective therapy is warranted to prevent disease progression. Aside from weight loss and lifestyle modifications, pharmacologic treatments remain limited. In line with this, the role of a novel oral hypoglycemic agent called sodium–glucose cotransporter 2 (SGLT-2) inhibitor on the treatment of NAFLD has recently been investigated in various animal studies done on rodents models^8–10^ and human clinical trials^11,12^, with potential positive effects.

In a real-world study of 56 patients with NAFLD and Type 2 diabetes mellitus who received SGLT-2 inhibitors for 48 weeks, there were statistically significant decreases in both controlled attenuation parameter (CAP) from 312 dB/m at baseline to 280 dB/m at week 48, and liver stiffness measurement (LSM) from 9.1 kPa at baseline to 6.7 kPa at week 48^11^. Additionally, the SGLT-2 inhibitor group showed statistically significant reductions in body weight, alanine transaminase (ALT), uric acid, and Fibrosis-4 (FIB-4) index at week 48 in comparison to the non-SGLT-2 inhibitor group that received other oral hypoglycemic medications using the 1:1 propensity-score matched analysis^11^. Moreover, a meta-analysis of 10 randomized controlled trials with 573 participants showed that use of SGLT-2 inhibitors yielded statistically significant reductions in the levels of ALT, aspartate transaminase (AST), liver proton density fat fraction (MRI-PDFF), visceral fat mass area, and subcutaneous fat areas^12^.

The impetus for this systematic review and meta-analysis is to give a more precise estimate of effect and address variations with use of SGLT-2 inhibitors in the treatment of NAFLD to help guide clinical practice guideline development, with a renewed focus on the use of imaging and histopathology outcome measures as found in the up-to-date randomized controlled trials.

The objective of this meta-analysis is to evaluate the effectiveness of sodium–glucose cotransporter-2 (SGLT-2) inhibitors in improving hepatic steatosis and hepatic fibrosis using imaging biomarkers and histopathology in patients with non-alcoholic fatty liver disease.

## Methodology

This present study was conducted in accordance with preferred reporting items for systematic review (PRISMA) guidelines^13^ (See also Supplementary Table S1).

### Eligibility criteria

Studies were selected based on the following inclusion criteria: (1) randomized-controlled trials, regardless of blinding status, that include studies examining adult participants more than or equal to 18 years of age with diagnosed non-alcoholic fatty liver disease irrespective of other comorbidities with or without diabetes mellitus, obese or non-obese; (2) use of SGLT-2 inhibitors in the active experimental group and not restricted to a particular sub-type, dosage, frequency or duration; (3) for the comparators, studies with placebo or the usual standard of care as relevant controls.

Non-randomized clinical trials, cohort studies, case control studies or cross-sectional studies were excluded. There were no limitations regarding the type of setting and length of follow-up.

The primary outcomes included the controlled attenuation parameter (CAP) which is a method used to measure the degree of ultrasound attenuation by hepatic fat at the central frequency of FibroScan®. The CAP value is expressed in dB/m. The next primary outcome was the liver stiffness measurement (LSM) which refers to the non-invasive quantification of liver stiffness by transient elastography using FibroScan®. The LSM value is expressed as kilopascal or kPa. Another primary outcome of interest is the proportion of patients with at least one point improvement or one-stage reduction in the histological scores with respect to hepatic steatosis, hepatocellular ballooning, lobular inflammation, and fibrosis after treatment.

The secondary outcomes include the Fibrosis-4 (FIB-4) index which is a non-invasive scoring system from several laboratory tests that can predict significant hepatic fibrosis patients. It includes age (years), AST level (U/L), platelet count (10^9^/L) and ALT (U/L). Other imaging biomarkers of hepatic steatosis included in this study were the liver-spleen attenuation ratio as quantified by computed tomography, and the magnetic resonance imaging derived proton-density-fat-fraction (MRI-PDFF) which enables a quantitative assessment of liver fat over the entire liver.

### Information sources

Two independent reviewers (A.M.O.L., and J.A.P.) performed a comprehensive electronic database search via *PUBMED*^14^, *Cochrane Central Register of Controlled Trials*^15^, *Embase*^16^ and *ACP Journal Club*^17^, published from inception to 29 December 2023. Other unpublished trials and study registries were also sought after by scanning the ClinicalTrials.gov, ISRCTN Register, EU Clinical Trials Register and WHO ICTRP. The planned literature search was not restricted to the English language or publication date filter.

### Search strategy

The MEDLINE search strategy entered at PubMED database^14^ was the use of the following terms: ((((((((((SGLT-2 inhibitor) OR (dapagliflozin)) OR (empagliflozin)) OR (ipragliflozin)) OR (tofogliflozin)) OR (canagliflozin)) OR (licogliflozin)) OR (luseogliflozin)) OR (ertugliflozin)) AND (non-alcoholic fatty liver disease)) OR (NAFLD) with filters for randomized controlled trial. These search terms were also similarly adapted for use with other electronic bibliographic databases^15–17^. We also attempted to look for other related articles that were not identified by electronic searches.

### Selection process

Literature search results were imported to *EPPI-Reviewer Version 4.14.2.0* for data management to facilitate the screening process^18^. Two independent reviewers (A.M.O.L, and J.A.P.) identified potentially eligible studies through screening of titles and abstracts, and assessed if these met the inclusion criteria. Any duplicate records of the same report were removed. For any disagreements between the authors, consensus of which articles to screen for full text were resolved by discussion. Subsequently, two researchers (A.M.O.L, and J.A.P.) independently screened full-text articles and scrutinized their eligibility. Again, any disagreements over the eligibility of the studies were resolved by consensus.

### Data collection process

Two reviewers (A.M.O.L, and J.A.P.) independently extracted data from every report with the use of a standardized data collection form. The retrieved data were then compared. Any disagreements or discrepancies during the data collection process were resolved by reviewing the data again, and through a consensus discussion. A.M.O.L entered the data into *Cochrane’s RevMan Web*^19^, and again double checked for its completeness and accuracy. For missing data or unclear information, the reviewers attempted to correspond with the study investigators through their respective official email addresses.

### Data items

We collected the following data from each included study: title and first author with citation details, study design, study location, total study duration, type and number of participants, study inclusion and exclusion criteria, baseline patient characteristics, detailed description of the intervention and control (type of drug used, dosage, frequency, duration of treatment), the outcome of interest and results.

### Study risk of bias assessment

Two independent reviewers (A.M.O.L, and J.A.P.) assessed the risk of bias in included studies using the *Cochrane Risk of Bias 2.0 (RoB 2.0)* tool^20^. The domains in *RoB 2.0* include bias arising from the randomization process; bias due to deviations from intended interventions; bias due to missing outcome data; bias in measurement of the outcome; and bias in selection of the reported result. The judgment of overall risk-of-bias were subdivided into “low risk of bias”, “some concerns” or “high risk of bias.”^20^. Any discrepancies in risk assessment were settled through constructive discussions between authors.

### Effect measures

We used the unstandardized mean differences at 95% confidence interval for continuous data given the same measurement scales to assess outcomes. Additionally, we utilized the final measurement outcomes rather than the change-from-baseline outcomes as the latter was not reported in most of the studies and considering the difficulty to measure the outcomes precisely coupled with the skewness of the distribution^21^, hence the former was selected. For dichotomous data, we calculated the risk ratio (RR) and their 95% confidence interval.

### Synthesis methods

To decide which studies were eligible for each synthesis, structured approaches were done with tabulation and coding of the main characteristics of the population, intervention, and outcomes. The intervention component of each study was coded along two dimensions namely SGLT-2 inhibitor only or SGLT-2 inhibitor plus another drug. The comparison group was likewise categorized into placebo only or standard of treatment, pioglitazone, ursodeoxycholic acid, tenegliptin, glimepiride, liraglutide, metformin, and metformin plus pioglitazone.

During the data synthesis, we noticed some continuous data presented as median and interquartile range. In this scenario, if the respective corresponding author cannot be contacted, we convert the study data into the estimated mean and standard deviation using the *R-package ‘estmeansd 0.2.1’* for which was primarily referenced from the Mcgrath et al.^22^ 2020 DEPRESsion Screening Data (DEPRESSD) Collaboration. There were also a few study data highlighted as mean and standard error (SE) for which it was converted to standard deviation (SD) using *Revman Web* by *Cochrane*^19^ and computed as SE multiplied by the square root of the sample size. In cases where there is no available information on variability measures, we did not exclude the studies from the meta-analysis. Instead, we imputed the missing data by getting the average standard deviations (SD) from other studies in the same meta-analysis.

To synthesize results, a meta-analysis of effect estimates was done using the *Revman Web* by *Cochran*e^19^. The random-effects meta-analysis model was chosen under the assumption that the true intervention effects are related through a distribution. The choice of this model was based on the clinical and methodological diversity of the included studies, and the concern for small-study effects. For continuous data, the inverse variance statistical method was used; while for dichotomous outcomes, the Mantel–Haenszel method was employed in consideration for some studies with small sample sizes and/or lower event rates^21^.

Heterogeneity among studies was identified in terms of clinical, methodological, and statistical factors. For statistical heterogeneity, assessment was done using the *I*^2^ statistics, with an interpretation of *I*^2^ value of 30–60% representing moderate heterogeneity, 50–90% indicating substantial heterogeneity, and 75–100% interpreted as considerable heterogeneity^21^. Whenever significant heterogeneity was detected, we proceeded with sensitivity analysis by omitting studies that are judged to be at high risk of bias, or excluding studies that are deemed ineligible such as unpublished data, inadequate sample sizes, substantial variances in patient characteristics, methodology or intervention^21^.

### Reporting bias assessment

Funnel plot asymmetry was assessed for meta-analysis with at least 10 studies. It was interpreted by visual inspection and statistical tests using Egger regression and Begg & Mazumdar rank correlation. Additionally, assessment for selective outcome reporting or selective non-reporting of results was completed by directly examining if a study protocol/register is available prior to the published study, and if the outcomes stated in the protocol are concurrent with the published report. If a protocol was unavailable, we then compared the outcomes reported in the methods and results section of the published paper.

### Certainty assessment

Two independent reviewers (A.M.O.L. and J.A.P.) evaluated the certainty of evidence for each outcome using the Grades of Recommendation, Assessment, Development and Evaluation Working Group (GRADE)^23^ approach. The rating criteria for considering lowering or raising the certainty of evidence was dependent on these factors, which consisted of risk of bias, inconsistency, indirectness, imprecision, publication bias, large effect, dose response and other plausible confounding bias. We utilized the online *GRADEpro*^23^ to generate the summary of findings table together with the footnotes as rationale for up-grade or down-grade the certainty of evidence.

## Results

### Study selection

**Figure 1:**
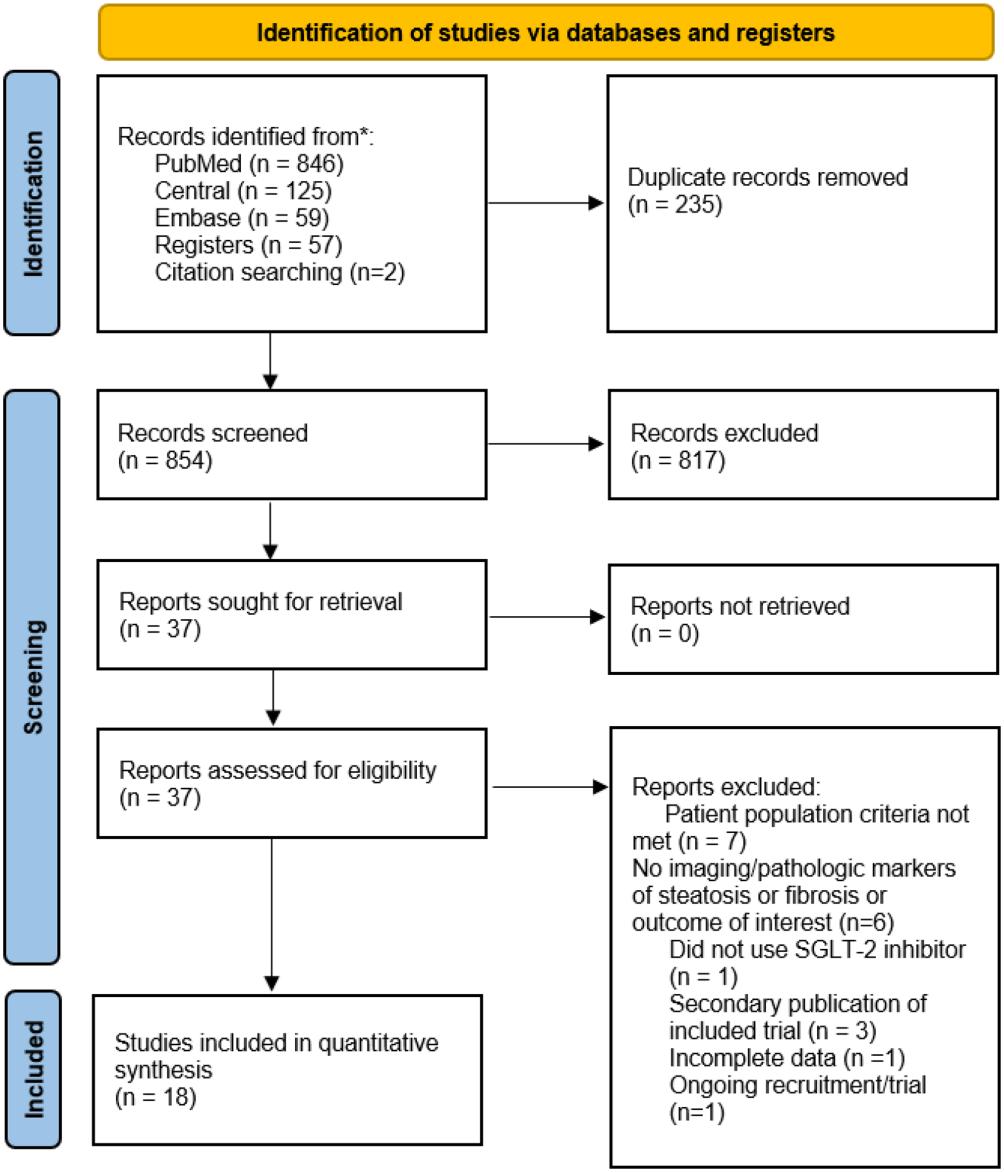
PRISMA 2020 flow diagram for systematic reviews.

In the initial database search, we found a total of 1089 potential records (see Fig. 1). After eliminating the duplicates, we proceeded with screening the 854 records, from which we reviewed 37 full-text articles, and finally, we included 18 randomized-controlled studies^24–41^. Also, we comprehensively searched the references and other documents which cited these studies. However, no additional articles that fulfilled inclusion criteria were found in these searches.

We excluded 19 studies from our review because of the following reasons: Population criteria was not met in seven studies; there were no imaging/pathologic markers of steatosis or fibrosis or outcome of interest in six studies; SGLT-2 inhibitor was not used in one study; there were secondary publications of three included studies; there was incomplete data in one study; and there was still ongoing recruitment/trial in one study.

### Study characteristics

Across 18 trials, a total of 1330 participants were included, with 631 in the SGLT-2 inhibitor group and 657 in the control group. No major differences in baseline characteristics were identified among the included studies. The mean age of participants ranged from 43.8 to 65.0 years in the SGLT-2i group and 41.8–65.6 years in the control group. All studies enrolled patients with non-alcoholic fatty liver disease based on ultrasonography^24,27,29,32,35–37,40^, computed tomography^32,33,35^, magnetic resonance imaging^28,34^, fatty liver index^26^, or pre-treatment biopsy findings^30,38,39^. Subsequently, two out of 18 studies excluded Type 2 diabetes mellitus in their eligibility criteria^37,40^. Empagliflozin, dapagliflozin, tofogliflozin, luseogliflozin, licogliflozin and ipragliflozin were the studied sodium-glucose cotransporter-2 inhibitors for the experimental group; whereas the control group had varied medications used including pioglitazone, metformin, glimepiride, tenegliptin, liraglutide, placebo or the usual standard of care. Lifestyle modifications and anti-diabetic drugs (including metformin, DPP4-inhibitors, sulfonylureas, and insulin) without any SGLT-2 inhibitors, pioglitazone or GLP-1 agonist, comprise the usual standard of care for Type 2 diabetes mellitus, which was used by three studies as control^34,36,38^. The course of treatment ranged from 12 to 72 weeks depending on the included trial. Other in-depth characteristics of included studies were presented in Table 1 and in the Supplementary Tables S2–S4 online.

**Table 1:** Characteristics of included studies.

Study author, year	Study design, location	Study population	Allocation ratio	Comparative treatment	Active Intervention(s)	Primary endpoint	Time frame
						Secondary endpoints	
Chehrehgosha, 2021	Prospective randomized, double-blind, placebo-controlled trial; Iran	Patients with NAFLD and T2DM	1:1:1	Placebo (n = 37)	Empagliflozin 10 mg (n = 35)	Change from baseline in controlled attenuation (CAP) score	24 weeks
				Pioglitazone 30 mg (n = 34)		Changes in liver stiffness measurement (LSM), liver enzymes, fasting insulin, HOMA-IR, VAT, body composition parameters, and non-invasive scores	
Cho, 2021	Multicenter, open-label, prospective, randomized, parallel-group comparison trial; Japan	Patients with NAFLD and T2DM	1:1	Pioglitazone 15–30 mg (n = 26)	Dapagliflozin 5 mg (n = 27)	Change in fatty liver index (FLI)	24 weeks
						Changes in liver enzymes, lipid and glycemic profile, FIB-4 index	
Chu, 2022	Single center, randomized, controlled trial	Patients with NAFLD and T2DM	1:1:1	Liraglutide 0.6–1.8 mg (n = 45)	Dapagliflozin 10 mg + Liraglutide 0.6–1.8 mg (n = 45)	Changes in hs-CRP, soluble interleukin-2 receptor, FBS, Hba1c, Tg, ALT, GGT, TBA	20 weeks
					Dapagliflozin 10 mg only (n = 45)	Changes in fatty liver index (FLI) and LSM	
Elhini, 2022	Randomized, double-blinded clinical study; Egypt	Patients with NAFLD and T2DM	1:1:1	Placebo (n = 80)	Empagliflozin 25 mg (n = 80)	Change from baseline in liver fat content (LFC) by MRI (proton density fat fraction [PDFF])	24 weeks
				Ursodeoxycholic acid 250 mg (n = 80)		Changes in liver enzymes, lipid and glycemic profiles, FIB-4 index, and non-alcoholic fatty liver score (NFS)	
Eriksson, 2018	Randomized placebo-controlled double-blind parallel-group study; Sweden	Patients with NAFLD and T2DM	1:1:1:1	Placebo (n = 21)	Combination of both Omega-3 carboxylic acids and Dapagliflozin (n = 22)	Change from baseline in liver fat content (LFC) by MRI-PDFF	12 weeks
				Dapagliflozin 10 mg (n = 21)			
				Omega-3 (n-3) carboxylic acids 4 g (n = 20)		Changes in total liver volume, markers of glucose and lipid metabolism as well as of hepatocyte injury and oxidative stress	
Han, 2020	Randomized, controlled, parallel, open-label study; Korea	Patients with NAFLD and T2DM	1:2	Metformin and Pioglitazone (n = 15)	Ipragliflozin 50 mg as an add-on treatment (n = 30)	Change in total visceral fat as measured by dual-energy x-ray absorptiometry (DXA)	24 weeks
						Changes in CAP, fatty liver index, and NAFLD fatty liver score Changes in SFA, VFA, SFA/VFA ratio, glycemic parameters, lipid profile, and liver enzymes	
Harrison, 2022	Multicenter, randomized, double-blind, placebo-controlled study; Argentina, Canada, Israel, the Netherlands, Russia, Taiwan, Thailand and the United States	Patients with NASH and T2DM	1:2:2	Placebo (n = 21)	Licogliflozin 150 mg (n = 43)	Change in serum alanine aminotransferase (ALT) levels	12 weeks
					Licogliflozin 30 mg (n = 43)	Changes in liver fat content (LFC), serum aspartate aminotransferase (AST), gamma-glutamyl transferase (GGT), anthropometric parameters, enhanced liver fibrosis score and its components, safety outcomes, metabolic parameters, lipid profile, and other liver fibrosis markers	
Hu, 2020	Single-center, randomized, controlled trial	Patients with NAFLD and T2DM	1:1	Metformin 500 mg TID (n = 30)	Dapagliflozin 5 mg (n = 30)	Changes from baseline in BMI, waist circumference, waist-to-hip ratio, SBP, DBP, lipid profile, BUA, ACR FBS, fasting insulin, HbA1C	12 weeks
						Changes from baseline in general indicators such as HOMA-IR, ALT, LSM and CAP	
Ito, 2017	Randomized, open-label, multicenter, active-controlled trial; Japan	Patients with NAFLD and T2DM	1:1	Pioglitazone 15–30 mg (n = 34)	Ipragliflozin 50 mg (n = 32)	Change from baseline in the liver-to-spleen attenuation (L/S) ratio by CT tomography	24 weeks
						Changes from baseline in AST, ALT, HbA1c, FPG, body weight, abdominal visceral fat area (VFA), and subcutaneous fat area (SFA) Changes in GGT, serum ferritin, serum type IV collagen 7S, NAFLD fibrosis score, FIB4 index, NAFIC score, HOMA-IR, Adipo-IR, lipid profiles, serum adiponectin, serum creatinine, eGFR, and blood pressure values	
Kinoshita, 2020	Prospective, open-label randomized, clinical study; Japan	Patients with NAFLD and T2DM	1:1:1	Pioglitazone 7.5–15 mg (n = 36)	Dapagliflozin 5 mg (n = 40)	Change from baseline in the liver-to-spleen attenuation (L/S) ratio by CT tomography	28 weeks
				Glimepiride 0.5–1 mg (n = 34)		Changes in hepatobiliary enzymes, bodyweight, visceral fat area (VFA), fasting plasma glucose, insulin, HbA1c, lipid profile, serum total adiponectin, type IV collagen 7S, and the fibrosis score (NAFLD fibrosis score, Fibrosis-4 index, NAFIC score)	
Kuchay, 2018	Prospective, open-label, randomized clinical study; India	Patients with NAFLD and T2DM	1:1	Standard treatment for Type 2 diabetes mellitus without SGLT-2 inhibitors, pioglitazone, GLP-1 agonist (n = 25)	Empagliflozin 10 mg (n = 25)	Change from baseline in liver fat content (LFC) by MRI-PDFF	20 weeks
						Change in serum AST, ALT, and GGT levels	
Shibuya, 2017	Single-center, prospective, randomized, open-label, controlled study; Japan	Patients with NAFLD and T2DM	1:1	Metformin 1500 mg (n = 16)	Luseogliflozin 2.5 mg (n = 16)	Change from baseline in the liver-to-spleen attenuation (L/S) ratio by CT tomography	24 weeks
						Changes in VFA, FPG, BMI, HBa1c, ALT, C-peptide immunoreactivity, and CPR index	
Shimizu, 2018	Prospective, randomized, open-label, blinded endpoint design; Japan	Patients with NAFLD and T2DM	1:1	Standard treatment for Type 2 diabetes mellitus without SGLT-2 inhibitors, pioglitazone, GLP-1 agonist (n = 28)	Dapagliflozin 5 mg (n = 35)	Change from baseline in controlled attenuation (CAP) score	24 weeks
						Changes in LSM, HbA1c, VAT, liver enzymes, and various markers and scores for hepatic fibrosis	
Taheri, 2020	Prospective, randomized, double blind, placebo-controlled, clinical trial; Iran	Patients with NAFLD but without T2DM	1:1	Placebo (n = 47)	Empagliflozin 10 mg (n = 43)	Change from baseline in controlled attenuation (CAP) score	24 weeks
						Change in LSM, liver enzymes, fasting insulin, HOMA2, grade of fatty liver by ultrasound, visceral adipose tissue (VAT), and other DXA parameters, and various laboratory scores for hepatic fibrosis	
Takahashi, 2021	Multicenter, randomized, controlled trial; Japan	Patients with NAFLD and T2DM	1:1	Lifestyle modifications, including diet and exercise therapy, and/or took antidiabetic drugs, without SGLT2 inhibitors, pioglitazone, or GLP-1 agonist (n = 28)	Ipragliflozin 50 mg (n = 27)	Changes in glycemic profile, BMI, and liver enzymes Changes in pathological findings between the first and second liver biopsies	72 weeks
Takeshita, 2022	Randomized, open-label, parallel-group trial; Japan	Patients with NAFLD and T2DM	1:1	Glimepiride 0.5–6 mg (n = 20)	Tofogliflozin 20 mg (n = 20)	Percentage of participants with at least 1 point improvement in each histological score of steatosis, hepatocellular ballooning, lobular inflammation, and fibrosis	48 weeks
						Changes in liver enzymes, metabolic markers, and hepatic gene expression profiles	
Tobita, 2022	Double-blind randomized prospective study; Japan	Patients with NAFLD but without T2DM	1:1	Teneligliptin 20 mg (n = 10)	Dapagliflozin 5 mg (n = 12)	Alanine aminotransferase (ALT) reduction level	12 weeks
						Changes in liver enzymes, lipid and glycemic profile, body composition, blood pressure and hand group strength	
Yoneda, 2021	Open-label, prospective, single-center, randomized clinical trial; Japan	Patients with NAFLD and T2DM	1:1	Pioglitazone 15–30 mg (n = 19)	Tofogliflozin 20 mg (n = 21)	Change from baseline in liver fat content (LFC) by MRI-PDFF	24 weeks
						Changes in ALT levels, adverse events (AEs), results of standard laboratory analysis, physical examination, and vital signs	

NAFLD, non-alcoholic fatty liver disease; T2DM, Type 2 Diabetes mellitus; CAP, controlled attenuation parameter; LSM, liver stiffness measurement; FLI, fatty liver index; FIB-4, fibrosis-4 index score; MRI-PDFF, magnetic resonance imaging-proton density fat fraction; L/S ratio, liver-to-spleen attenuation ratio; FPG, fasting plasma glucose; HBa1c, glycosylated hemoglobin; LFC, liver fat content; VFA, visceral fat area; SFA, subcutaneous fat area; VAT, visceral adipose tissue; BMI, body mass index; HOMA-IR, Homeostatic Model Assessment for Insulin Resistance; DXA, dual-energy x-ray absorptiometry; ALT, alanine aminotransferase; AST, aspartate aminotransferase; GGT, gamma-glutamyl transferase.

### Risk of bias in studies

The risk of bias of individual trials were assessed using the *RoB 2.0 tool*^20^. A summary of these assessments is provided in Table 2. Overall, most of the included studies (16/18) presented some concerns for risk of bias, while two studies were assessed as having low risk of bias.

**Table 2:** Summarized RoB 2.0 of included studies.

Study	Bias arising from the randomization process	Bias due to deviations from intended interventions	Bias due to missing outcome data	Bias in measurement of the outcome	Bias in selection of the reported result	Overall risk of bias
Chehrehgosha, 2021	Low risk	Low risk	Low risk	Low risk	Low risk	Low risk
Cho, 2021	Low risk	Some concerns	Low risk	Low risk	Low risk	Some concerns
Chu, 2022	Some concerns	Low risk	Low risk	Low risk	Low risk	Some concerns
Elhini, 2022	Low risk	Some concerns	Some concerns	Low risk	Low risk	Some concerns
Eriksson, 2018	Low risk	Some concerns	Low risk	Low risk	Low risk	Some concerns
Han, 2020	Some concerns	Low risk	Low risk	Some concerns	Low risk	Some concerns
Harrison, 2022	Low risk	Some concerns	Some concerns	Low risk	Low risk	Some concerns
Hu, 2020	Some concerns	Low risk	Low risk	Low risk	Low risk	Some concerns
Ito, 2017	Some concerns	Low risk	Low risk	Some concerns	Low risk	Some concerns
Kinoshita, 2020	Low risk	Some concerns	Low risk	Low risk	Low risk	Some concerns
Kuchay, 2018	Some concerns	Some concerns	Low risk	Low risk	Low risk	Some concerns
Shibuya, 2017	Low risk	Low risk	Low risk	Low risk	Low risk	Low risk
Shimizu, 2018	Some concerns	Some concerns	Low risk	Low risk	Low risk	Some concerns
Taheri, 2020	Some concerns	Some concerns	Low risk	Low risk	Low risk	Some concerns
Takahashi, 2021	Some concerns	Some concerns	Low risk	Low risk	Low risk	Some concerns
Takeshita, 2022	Some concerns	Low risk	Low risk	Low risk	Low risk	Some concerns
Tobita, 2022	Low risk	Some concerns	Low risk	Low risk	Low risk	Some concerns
Yoneda, 2021	Low risk	Some concerns	Low risk	Low risk	Low risk	Some concerns

### Results of individual studies

**Figure 2:**
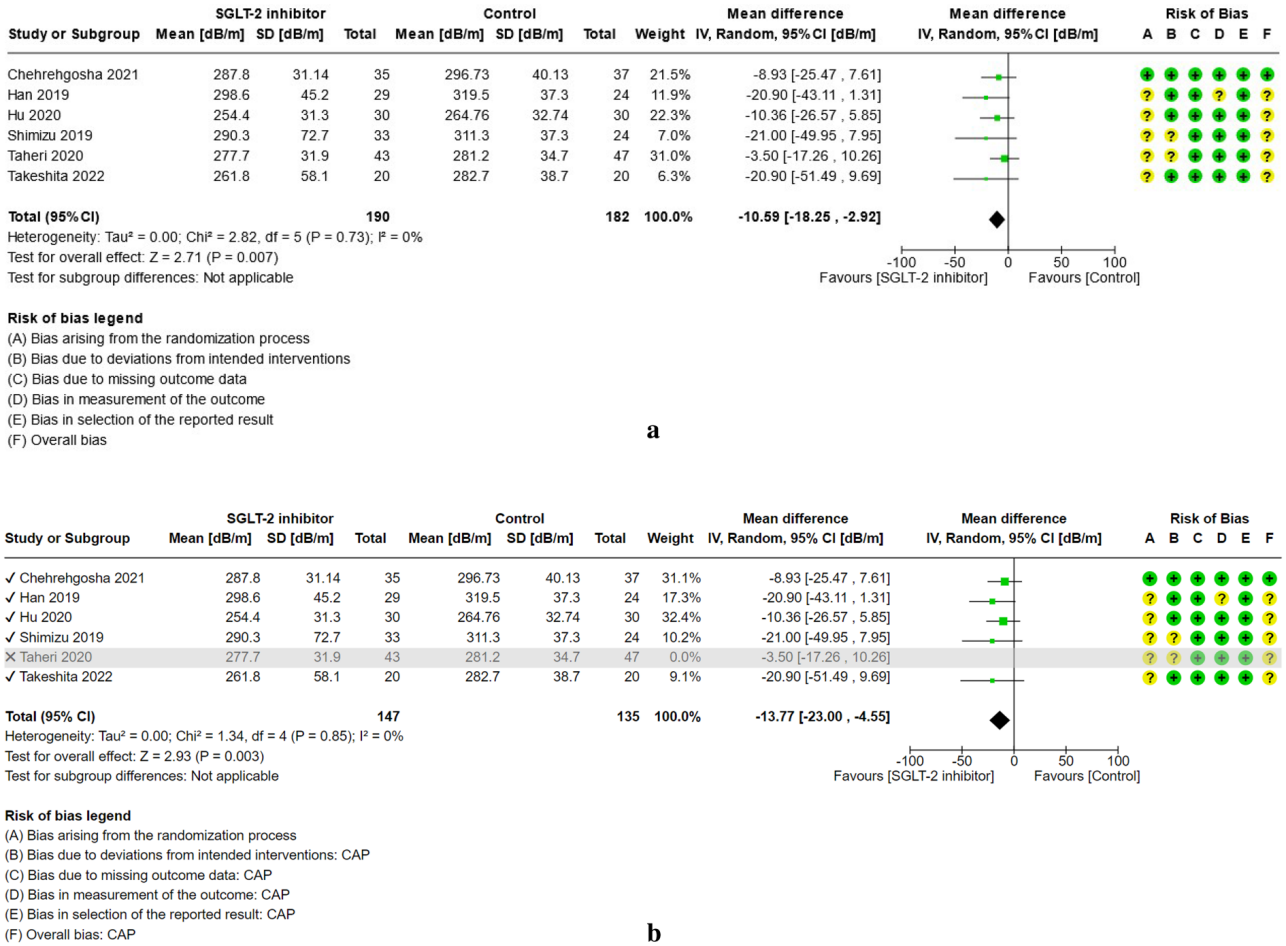
(**a**) Forest plot of comparison: summary of mean difference in controlled attenuation parameter (CAP) post-treatment in patients with NAFLD randomized to either SGLT-2 inhibitor or control. (**b**) Forest plot of comparison [Sensitivity analysis, Taheri 2020 excluded]: summary of mean difference in controlled attenuation parameter (CAP) post-treatment in patients with NAFLD randomized to either SGLT-2 inhibitor or control.

**Figure 3:**
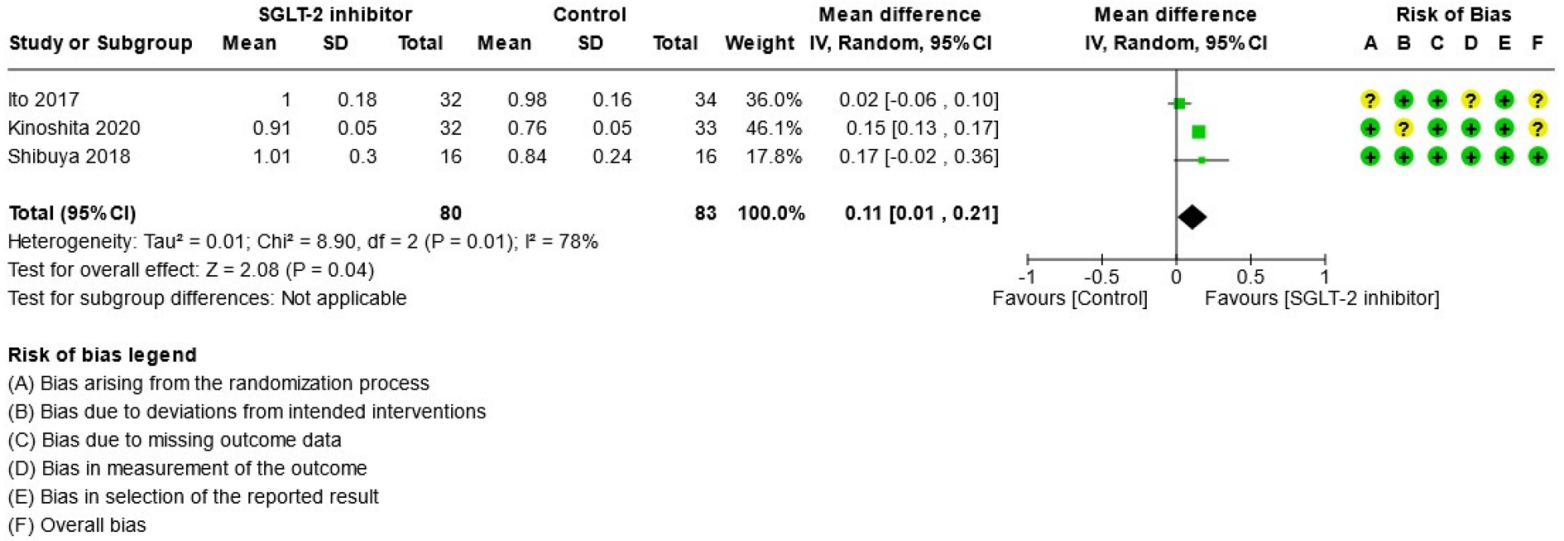
Forest plot of comparison: summary of mean difference in liver-to-spleen attenuation (L/S) ratio post-treatment in patients with NAFLD randomized to either SGLT-2 inhibitor or control. *Shibuya 2018 median and interquartile range study data converted to mean and SD using R-package ‘estmeansd 0.2.1’.

**Figure 4:**
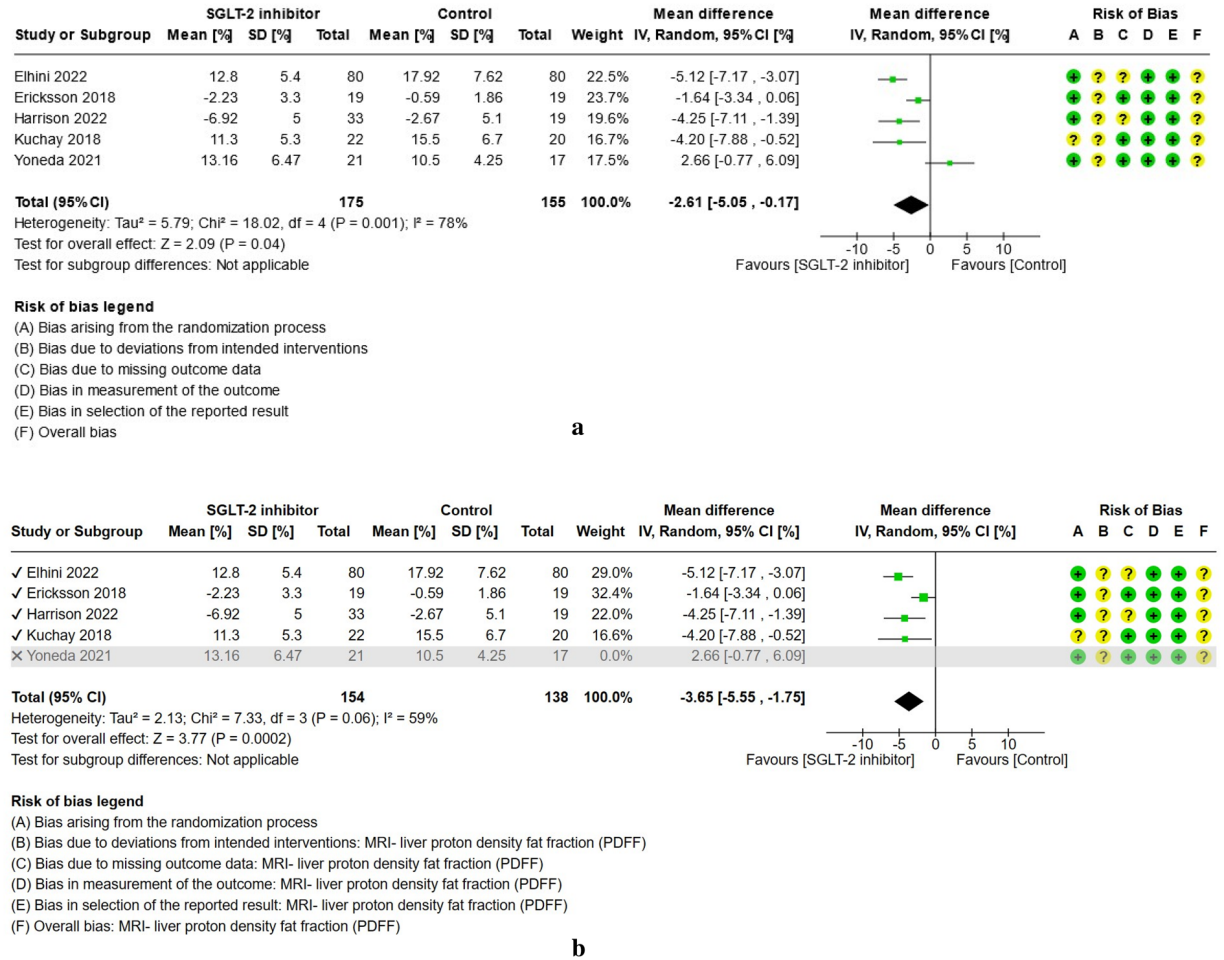
(**a**) Forest plot of comparison: summary of mean difference in liver proton density fat fraction (PDFF) post-treatment in patients with NAFLD randomized to either SGLT-2 inhibitor or control. *Harrison 2022 study—standard deviation derived from standard error calculated by Revman. **Ericksson 2018 and Harrison 2022 study measured as change-from-baseline scores. (**b**) Forest plot of comparison [Sensitivity analysis, Yoneda 2021 excluded]: summary of mean difference in liver proton density fat fraction (PDFF) post-treatment in patients with NAFLD randomized to either SGLT-2 inhibitor or control. *Harrison 2022 study—standard deviation derived from standard error calculated by Revman. **Ericksson 2018 and Harrison 2022 study measured as change-from-baseline score.

**Figure 5:**
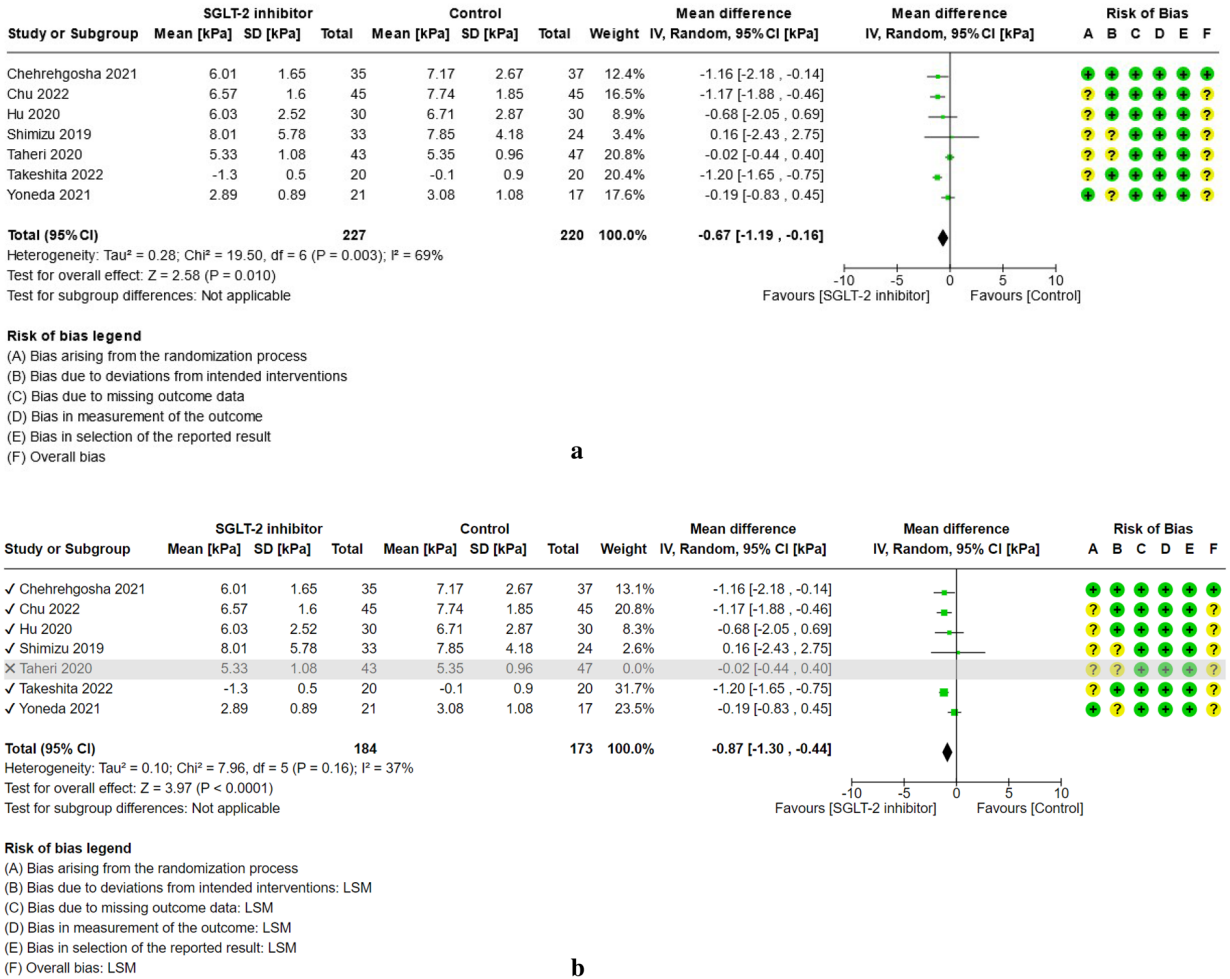
(**a**) Forest plot of comparison: summary of mean difference in liver stiffness measurement (LSM) post-treatment in patients with NAFLD randomized to either SGLT-2 inhibitor or control. *Takeshita 2022 measured as change-from-baseline scores. (**b**) Forest plot of comparison [Sensitivity analysis, Taheri 2020 excluded]: summary of mean difference in liver stiffness measurement (LSM) post-treatment in patients with NAFLD randomized to either SGLT-2 inhibitor or control. *Takeshita 2022 measured as change-from-baseline scores.

**Figure 6:**
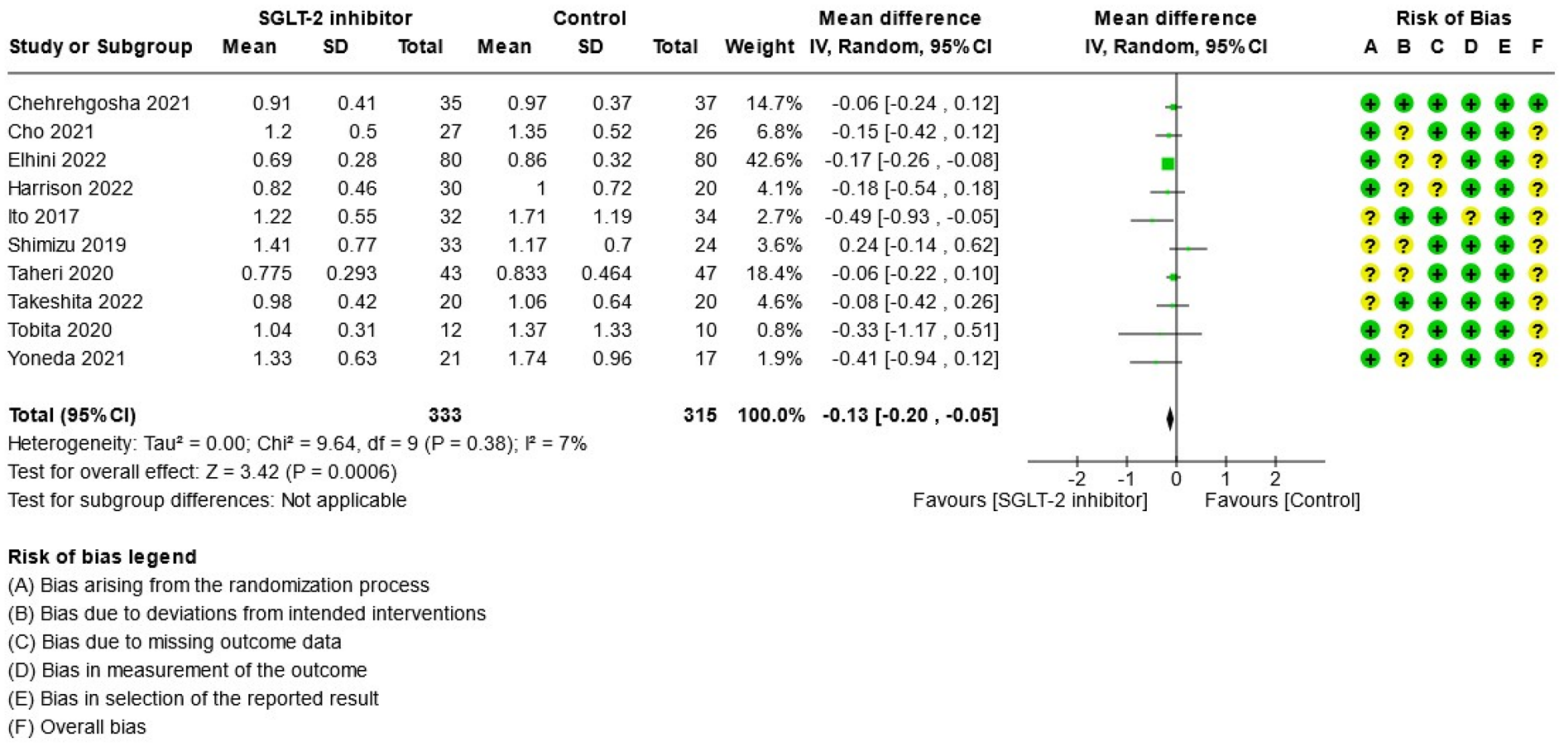
Forest plot of comparison: summary of mean difference in FIB-4 index post-treatment in patients with NAFLD randomized to either SGLT-2 inhibitor or control. *Takeshita 2022 median and interquartile range study data converted to mean and SD using R-package ‘estmeansd 0.2.1’.**Harrison 2022 with imputed standard deviation.

**Figure 7:**
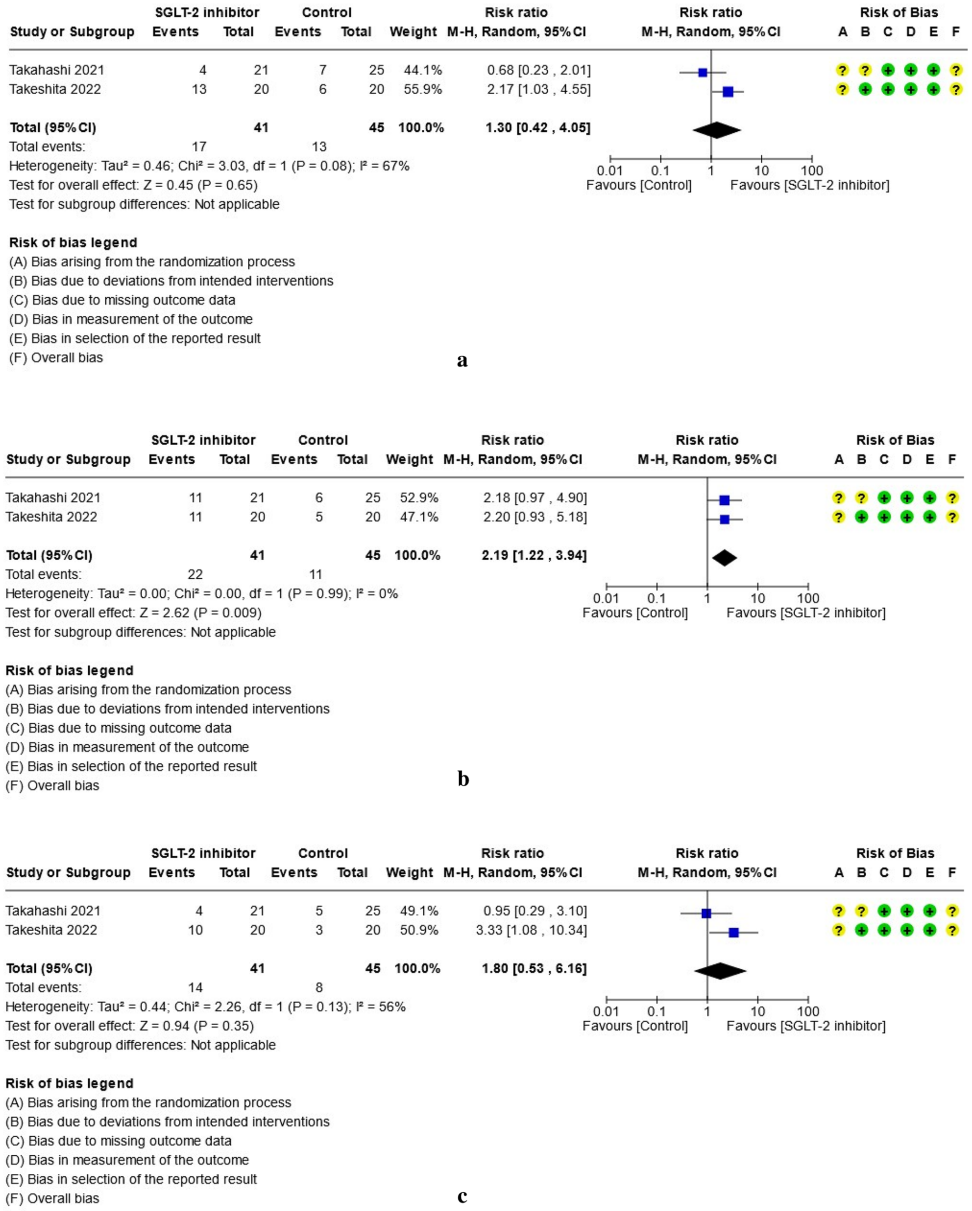

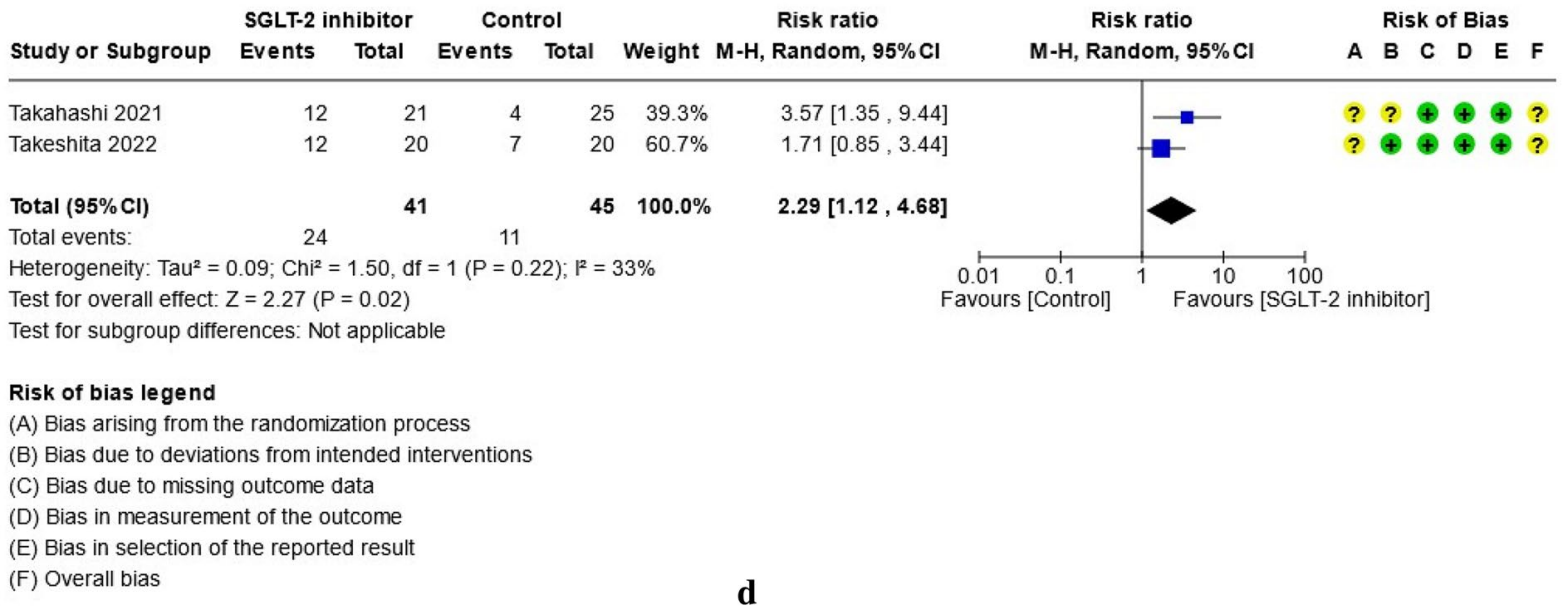
(**a**) Forest plot of comparison: summary of one-score or one-stage reduction in hepatic steatosis after treatment in patients with NAFLD randomized to either SGLT-2 inhibitor or control. (**b**) Forest plot of comparison: summary of one-score or one-stage reduction in hepatocellular ballooning after treatment in patients with NAFLD randomized to either SGLT-2 inhibitor or control. (**c**) Forest plot of comparison: summary of one-score or one-stage reduction in lobular inflammation after treatment in patients with NAFLD randomized to either SGLT-2 inhibitor or control. (**d**) Forest plot of comparison: Summary of one-score or one-stage reduction in liver fibrosis after treatment in patients with NAFLD randomized to either SGLT-2 inhibitor or control.

For study results, see Figs. 2, 3, 4, 5, 6 and 7 which present the summary statistics for each of the individual studies, and the pooled effect estimates with their confidence intervals using Forest plots. The risk of bias judgments was also displayed beside the plot to consider the limitations of interpreting the findings.

### Results of syntheses

#### Effect on hepatic steatosis

##### Controlled attenuation parameter (CAP)

Six randomized controlled trials (RCTs)^24,29,31,36,37,39^, including a combined total of 372 patients, compared the mean difference in controlled attenuation parameter (CAP) among non-alcoholic fatty liver patients given SGLT-2 inhibitor or the control intervention (see Fig. 2a). Each study utilized the transient liver elastography via FibroScan® to measure the controlled attenuation parameter as assessment for liver fat content. Five of the trials had some concerns for bias overall, owing to the lack of information on allocation concealment and/or use of per-protocol analysis. Empagliflozin was administered in two of the trials^24,37^, while ipragliflozin, tofogliflozin, and dapagliflozin were used in other trials as the active experimental group. For the control group, the medication given in each study slightly varies. Four trials were given standard of treatment for Type 2 diabetes mellitus, including one study receiving glimepiride^39^, metformin only^31^, respectively; the latter as combined metformin and pioglitazone^29^; whereas the other remaining two studies were given placebo^24,37^. After combining the results, the pooled mean difference in the reduction of CAP scores after randomly assigning to SGLT-2 inhibitor treatment versus the other comparators was (MD: − 10.59 dB/m, 95% CI [− 18.25, − 2.92], p = 0.007, I^2^ = 0%, moderate certainty of evidence).

On sensitivity analysis, the study by Taheri et al.^37^ was excluded as the sample population criteria did not include NAFLD patients with Type 2 diabetes mellitus (see Fig. 2b). As compared with the previous pooled result, findings showed a higher mean difference in the reduction of CAP scores (MD: − 13.77 dB/m, 95% CI [− 23.00, − 4.55], p = 0.003, I^2^ = 0%, moderate certainty of evidence).

##### Liver-to-spleen attenuation (L/S) ratio

Only three studies^32,33,35^ measured the degree of fatty liver using L/S ratio on CT tomography during the study period (see Fig. 3). The liver-to-spleen attenuation ratio was calculated as the average liver attenuation at the region of interest divided by the average spleen attenuation using the unenhanced CT tomography. In total, 80 patients received SGLT-2 inhibitors and 83 patients received the control intervention. One trial^35^ was assessed to have low risk of bias while the remaining two^32,33^ had some concerns for bias due to lack of information on allocation concealment or blinding of the outcome assessor. The results showed that the SGLT-2 inhibitors significantly increased the L/S ratio by weighted mean difference (0.11, 95% CI [0.01, 0.21], p = 0.04, I^2^ = 78%, low certainty of evidence) compared to the control group.

##### Liver proton density fat fraction (PDFF)

Five clinical trials^27,28,30,34,41^ provided data on the liver proton density fat fraction (PDFF) using magnetic resonance imaging (see Fig. 4a). A total of 175 cases and 155 controls had measured the MRI-PDFF (%) over the study period. The types of SGLT-2 inhibitors used include empagliflozin, dapagliflozin, licogliflozin, and tofogliflozin. The control intervention group consisted of placebo or standard of treatment for Type 2 diabetes mellitus other than SGLT-2 inhibitor, or pioglitazone treatment. Most of the studies presented some concerns for bias due to deviations from intended interventions with use of per-protocol analysis, and one trial^34^ lacked details on the concealment of the allocation sequence. The meta-analysis showed a trend towards a reduction in the liver proton density fat fraction at the end of treatment (MD: − 2.61%, 95% CI [− 5.05, − 0.17], p = 0.04, I^2^ = 78%, low certainty of evidence). In the sensitivity analysis, the study by Yoneda et al.^41^ was excluded owing to its control group being given pioglitazone, which is a medication shown to have some evidence in the treatment of NAFLD. On the other hand, the other trials consisted of placebo or standard of treatment groups. Results showed a consistent finding in the difference in means in the liver proton density fat fraction between SGLT-2 inhibitors and control with note of a decrease in heterogeneity (MD: − 3.65%, 95% CI [− 5.55, − 1.75], p = 0.0002, I^2^ = 59%, low certainty of evidence) (see Fig. 4b).

#### Effect on hepatic fibrosis

##### Liver stiffness measurement (LSM)

Analysis on the effect of SGLT-2 inhibitors versus control on the decrease in liver stiffness measurement (LSM) using meta-analysis is provided in Fig. 5a. Across seven randomized controlled trials (RCTs)^24,26,31,36,37,39,41^, a total of 227 patients in the SGLT-2 group, and 220 patients in the control group were included. Only one trial^37^ excluded Type 2 diabetes mellitus in the non-alcoholic fatty liver disease population. All trials measured LSM using transient liver elastography or FibroScan® except for one trial^40^ which used magnetic resonance elastography (MRE). Two studies^24,37^ used empagliflozin as the SGLT-2 inhibitor while the other two trials^39,41^ received tofogliflozin, and the remaining ones had dapagliflozin^31,36^, or dapagliflozin plus liraglutide as intervention^26^. The control groups were given other standard Type 2 diabetes mellitus treatments excluding SGLT-2 inhibitor, or placebo with moderate intensity physical activity and standard dietary advice only^37^. Results showed that the SGLT-2 inhibitors slightly decrease the LSM level by mean difference (− 0.67 kPa, 95% CI [− 1.19, − 0.16], p = 0.010, I^2^ = 69%, low certainty of evidence) compared to comparators. After performing a sensitivity analysis for which the study by Taheri et al.^37^ was excluded due to its non-inclusion of Type 2 diabetes mellitus population, a trend was observed towards a moderate reduction in the LSM level with further decrease in heterogeneity (MD: − 0.87 kPa, 95% CI [− 1.30, − 0.44], p < 0.0001, I^2^ = 37%, low certainty of evidence) (see Fig. 5b).

##### Fibrosis-4 index (FIB-4)

The FIB-4 index is a simple and valuable non-invasive tool in estimating the risk of liver fibrosis. The compiled studies calculated it using the following formula: FIB-4 index = (Age [years] × AST [U/L])/(platelet [109/L] × ALT [U/L]). The mean baseline FIB-4 index across 10 studies^24,25,27–29,37,39–41^ ranges from 0.775 to 1.50 in SGLT-2 group, and 0.826–2.12 in the control group. Almost all studies have some concern for bias except for one study^24^ with low risk of bias. The pooled estimate demonstrated that SGLT-2 inhibitor treatment significantly decreased the FIB-4 index as compared to control (MD: − 0.12, 95% CI [− 0.21, − 0.04], p = 0.005, I^2^ = 16%, moderate certainty of evidence) (see Fig. 6).

#### Effect on histopathology (steatosis, hepatocellular ballooning, lobular inflammation, liver fibrosis)

Only two trials^39,40^ histologically evaluated all biopsied specimens at baseline and post-treatment at 48 and 72 weeks respectively with a combined total of 41 patients in the SGLT-2 group and 45 patients in the control group. The biopsied liver tissues were scored for hepatic steatosis, hepatocellular ballooning, and lobular inflammation using the NAFLD activity score (NAS)^42^ while the liver fibrosis was classified according to Brunt et al.^43^ criteria. The pathological outcomes for each category were summarized separately in Fig. 7a–d. In the meta-analysis, the SGLT-2 inhibitor group was noted to have a higher likelihood of having at least a one-score or one-stage reduction after treatment than the control group with respect to hepatocellular ballooning (RR: 2.19, 95% CI [1.22, 3.94], p = 0.009, I^2^ = 0%, moderate certainty of evidence) and liver fibrosis (RR: 2.29, 95% CI [1.12, 4.68], p = 0.02, I^2^ = 33%, moderate certainty of evidence). There were no significant differences identified between the two groups with respect to the changes in steatosis or lobular inflammation.

#### Reporting biases in syntheses

**Figure 8:**
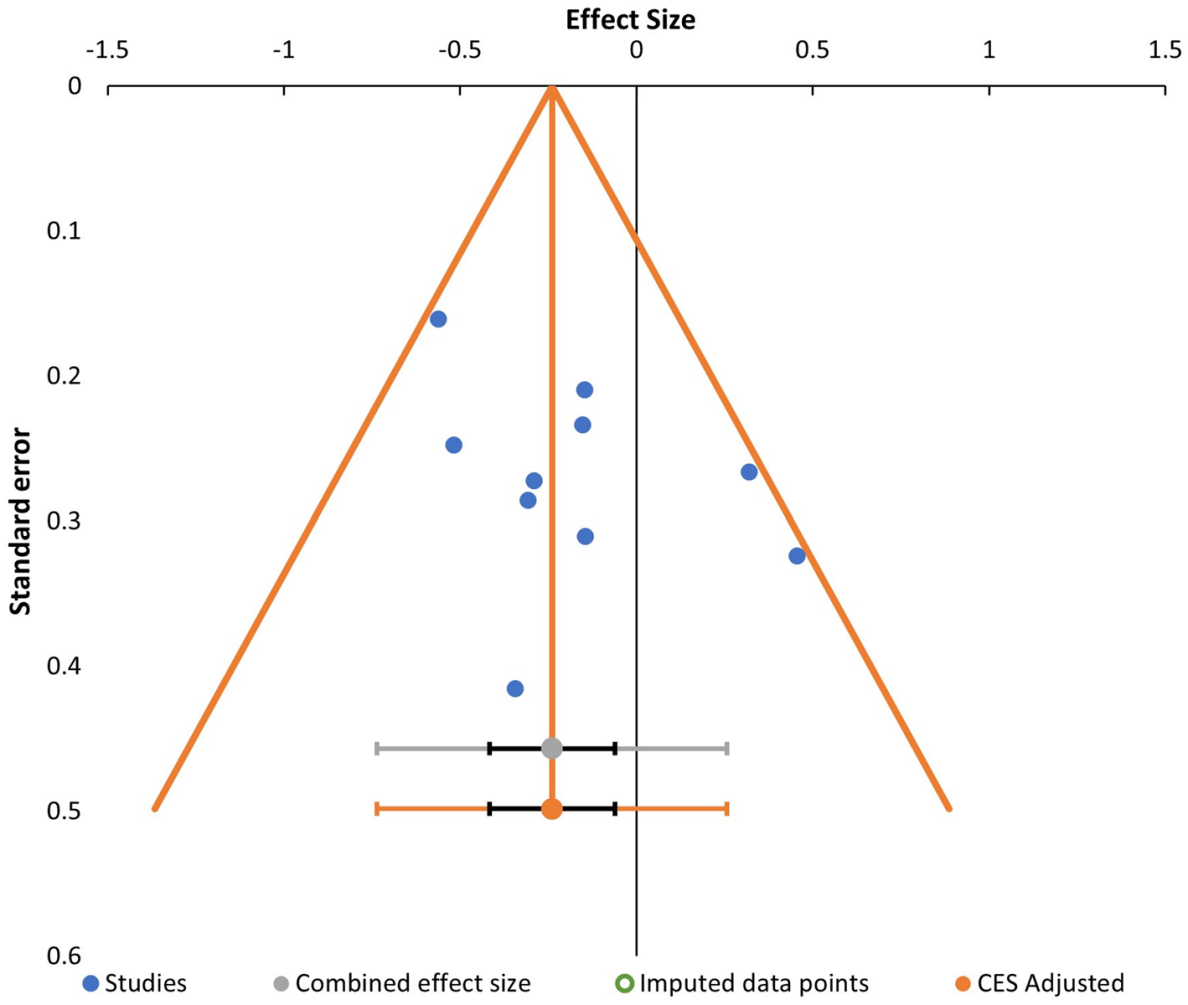
Funnel plot of the meta-analysis of studies based from FIB-4 index syntheses.

The funnel plot for Fig. 8 shows no evidence for publication bias. Egger’s test for a regression intercept gave a p-value of 0.129, while Begg and Mazumdar’s test for rank correlation gave a p-value of 0.655, indicating no publication bias.

All outcomes were found to be stated in their respective protocols and reported in the final publication reports. Hence, no selective outcome reporting was identified.

#### Certainty of evidence

Using the GRADE approach^23^, these outcomes, which consist of CAP, FIB-4 index, one-score reduction in hepatic steatosis and fibrosis, were noted to have moderate certainty of evidence. Others were designated as having low certainty of evidence given the inconsistency of results and imprecision which downgraded the evidence level. Other detailed summaries of findings were presented in Table 3, with footnotes explaining judgments.

**Table 3:** Summary of findings table by GRADEpro.

SGLT-2 inhibitor compared to Control for Non-alcoholic fatty liver disease (NAFLD)						
Patient or population: Non-alcoholic fatty liver disease Setting: Outpatient Intervention: SGLT-2 inhibitor Comparison: Control						
Outcomes	Anticipated absolute effects* (95% CI)		Relative effect (95% CI)	№ of participants (studies)	Certainty of the evidence (GRADE)	Comments
	Risk with Control	Risk with SGLT-2 inhibitor				
Controlled Attenuation Parameter	The mean controlled Attenuation Parameter was 292.698 dB/m	MD 10.59 dB/m lower (18.25 lower to 2.92 lower)	–	372 (6 RCTs)	⨁⨁⨁◯ Moderate^a^	SGLT-2 inhibitor likely results in a slight reduction in controlled attenuation parameter
Liver Stiffness Measurement	The mean liver Stiffness Measurement was 6.3167 kPa	MD 0.67 kPa lower (1.19 lower to 0.16 lower)	–	447 (7 RCTs)	⨁⨁◯◯ Low^a,b^	SGLT-2 inhibitor may result in a slight reduction in liver stiffness measurement
FIB-4 index	The mean FIB-4 index was 1.2063	MD 0.13 lower (0.2 lower to 0.05 lower)	–	598 (9 RCTs)	⨁⨁⨁◯ Moderate^c^	SGLT-2 inhibitor likely reduces FIB-4 index slightly
MRI- PDFF	The mean MRI- PDFF was 14.64%	MD 2.61% lower (5.05 lower to 0.17 lower)	–	330 (5 RCTs)	⨁⨁◯◯ Low^a,d^	The evidence suggests SGLT-2 inhibitor results in a slight reduction in MRI-PDFF (%)
L/S ratio	The mean L/S ratio was 0.86	MD 0.11 higher (0.01 higher to 0.21 higher)	–	163 (3 RCTs)	⨁⨁◯◯ Low^e,f^	The evidence suggests SGLT-2 inhibitor increases L/S ratio slightly
One-score reduction in hepatic steatosis (via histopathology)	289 per 1000	376 per 1000 (121 to 1000)	RR 1.30 (0.42 to 4.05)	86 (2 RCTs)	⨁⨁◯◯ Low^a,g^	SGLT-2 inhibitor may result in little to no difference in one-score reduction in hepatic steatosis
One-score reduction in hepatocellular ballooning (via histopathology)	244 per 1000	535 per 1000 (298 to 963)	RR 2.19 (1.22 to 3.94)	86 (2 RCTs)	⨁⨁⨁◯ Moderate^a^	SGLT-2 inhibitor likely results in one-score reduction in hepatocellular ballooning
One-score reduction in lobular inflammation (via histopathology)	178 per 1000	320 per 1000 (94 to 1000)	RR 1.80 (0.53 to 6.16)	86 (2 RCTs)	⨁⨁◯◯ Low^a,g^	SGLT-2 inhibitor may result in little to no difference in one-score reduction in lobular inflammation
One-score reduction in fibrosis (via histopathology)	244 per 1000	560 per 1000 (274 to 1000)	RR 2.29 (1.12 to 4.68)	86 (2 RCTs)	⨁⨁⨁◯ Moderate^a^	SGLT-2 inhibitor likely results in one-score reduction in fibrosis

*The risk in the intervention group (and its 95% confidence interval) is based on the assumed risk in the comparison group and the relative effect of the intervention (and its 95% CI).

**CI:** confidence interval; **MD:** mean difference; **RR:** risk ratio.

**GRADE Working Group grades of evidence.**

**High certainty:** we are very confident that the true effect lies close to that of the estimate of the effect.

** Moderate certainty:** we are moderately confident in the effect estimate: the true effect is likely to be close to the estimate of the effect, but there is a possibility that it is substantially different.

** Low certainty:** our confidence in the effect estimate is limited: the true effect may be substantially different from the estimate of the effect.

** Very low certainty:** we have very little confidence in the effect estimate: the true effect is likely to be substantially different from the estimate of effect.

**Explanations.**

^a^Imprecision due to widened confidence interval.

^b^Serious inconsistency with large heterogeneity I^2^ = 69%, *p* = 0.003.

^c^The lower bound value in the 95% CI is 0.05 which is close to no effect.

^d^Serious inconsistency with large heterogeneity I^2^ = 78%, *p* = 0.001.

^e^Serious inconsistency with large heterogeneity I^2^ = 78%, *p* = 0.01.

^f^The lower bound value in the 95% CI is 0.01 which is close to no effect.

^g^Serious inconsistency as results were not consistent across studies and the small number of trials limited the ability to draw a plausible conclusion.

## Discussion

The efficacy of SGLT-2 inhibitors in treating hepatic steatosis and fibrosis utilizing several imaging biomarkers and histopathology in patients with non-alcoholic fatty liver disease was investigated in this systematic review and meta-analysis of sixteen randomized controlled trials.

Consolidating the findings above, this meta-analysis found significant mean differences in CAP, L/S ratio, and MRI-PDFF after treatment, favoring the effect of SGLT-2 inhibitor over that of control especially in NAFLD patients.

These translate to a probable or slight positive effect on hepatic steatosis. In contrast, no significant differences were identified between the two groups with regards to the changes in steatosis or lobular inflammation on biopsy post-treatment. Nonetheless, interpretation of such findings in the latter appears to be inconclusive given the small number of studies.

With regards to hepatic fibrosis, there was a slight reduction in the LSM and FIB-4 index with use of SGLT-2 inhibitors in comparison to controls among non-alcoholic fatty liver patients with Type 2 diabetes mellitus. This was also confirmed histologically with the meta-analysis results showing at least a one-stage reduction in the treatment group with respect to hepatocellular ballooning and liver fibrosis.

Overall, our findings appear to be in line with those of other earlier systematic reviews and meta-analyses studies which also investigated the effects of SGLT-2 medications on NAFLD patients. A systematic review of four RCTs and four observational studies done by Raj et al.^44^ reported favorable effects of SGLT-2 inhibitors on the level of liver enzymes, liver fat, and fibrosis in patients with NAFLD. Similar positive effects of SGLT-2 inhibitors on liver enzyme and fat levels were also seen in individuals with NAFLD, according to a comprehensive evaluation of seven systematic reviews conducted by Shao et al.^45^.

A meta-analysis of four randomized trials evaluating liver fat content (LFC) using MRI by Coelho et al.^46^ (2020) showed a decrease in hepatic steatosis with the use of SGLT-2 inhibitors (MD: − 3.39%, 95% CI [− 6.01, − 0.77], p = 0.01, I^2^ = 89%). Moreover, a meta-analysis of two trials by Xing et al.^47^ reported a reduction in MRI-PDFF with the treatment of SGLT-2 inhibitors (MD: − 2.07%, 95% CI [− 3.86, − 0.28], p = 0.02, I^2^ = 10%). Another meta-analysis of two to four trials done by Song et al.^48^ revealed concordant findings, showing significant reduction in the level of liver controlled attenuation parameter (CAP) (MD: 0.29, 95% CI [− 26.95 to − 13.64], p < 0.00001, I^2^ = 0%), MRI-PDFF (MD:1.97, 95% CI [− 3.49 to − 0.45], p = 0.01, I^2^ = 11%), NAFLD score (MD: 0.55, 95% CI [1.04 to − 0.05], p = 0.03, I^2^ = 0%), fatty liver index (FLI) (MD: 11.21, 95% CI [− 16.53 to − 5.89], p < 0.0001, I^2^ = 0%), FIB‐4 index (MD: 0.25, 95% CI [− 0.39 to − 0.11], p = 0.0007, I^2^ = 10%), and increase in L/S ratio (MD: 0.16, 95% CI [0.10–0.22], p < 0.00001, I^2^ = 49%) with SGLT-2 inhibitor use.

In contrast, the meta-analysis done by Amjad et al.^49^ showed no statistically significant difference in fibrosis regression utilizing FIB-4 score (SMD = − 0.12, 95% CI: − 0.41 to 0.1, p = 0.994, I^2^ = 0%) and hepatic steatosis by using MRI-PDFF (SMD = − 0.31, 95% CI: –0.68 to 0.07, p = 0.502, I^2^ = 0%) between SGLT-2 inhibitors versus controls. However, it must be noted that this study only analyzed a total of three trials for both outcomes with inclusion of non-NAFLD population in one of the trials.

Despite the relevant findings in this meta-analysis, there were still some limitations in the evidence included in the review. We identified a few eligible studies with small sample sizes and inadequate power, leading to imprecise estimates. Also, most of the included studies had some concerns for risk of bias due to allocation concealment issues, use of per-protocol analysis, or missing results which partially affected the certainty (or confidence) in the body of evidence. Likewise, moderate to substantial statistical heterogeneity (I^2^ > 50%) was observed in some of the analysis which can be explained partially by variability in the studied participants, interventions and control used, in addition to the duration of treatment. Methodologically, different studies have diverse trial design methods (i.e., 1:1, 1:1:1, 2:1; double-blinded, open-label, and sample size differences) and quality which may reflect also in the heterogeneity measures. In terms of applicability to specific ethnic populations, majority of the studies included were conducted within Asia, with many subjects coming from Japan or China. The limited racial diversity within the study population may hence slightly affect the generalizability of the findings of this study.

Nonetheless, this updated meta-analysis was conducted in a comprehensive manner with an adequate number of databases searched by more than one reviewer to screen and extract data. Authors of the studies included were also contacted for completion and clarification of any missing information. The PRISMA guide & checklist^13^, and the Risk of bias 2.0 tool^20^ were likewise followed for transparency and completeness in reporting of the systematic review. Moreover, the certainty of evidence was presented using the GRADE approach^23^, which may assist physicians in the clinical decision-making process together with the patients.

As to implications for practice and policy, the positive evidence from this meta-analysis considers the use of SGLT-2 inhibitors for the treatment of Type 2 diabetes mellitus with an obese profile in the setting of concomitant non-alcoholic fatty liver disease.

The addition of SGLT-2 inhibitors to the usual management of dietary and lifestyle modifications in Type 2 diabetic patients with NAFLD may potentially prevent disease progression and the various complications that accompany the disease. Until now, there has been no standard clinically approved pharmacologic treatments for NAFLD. However, there is some growing evidence on the efficacy of other anti-diabetic medications including pioglitazone and GLP-1 agonists in its management. Although some studies included in this meta-analysis opted to compare the effects of SGLT-2 inhibitors with standard treatments not known to have any effects on NAFLD, some used pioglitazone^50^ or liraglutide^51^ as its control, which may slightly affect the positive magnitude of the results. Nevertheless, as a whole, the results of this meta-analysis remain useful in the clinical setting, thus providing an alternative treatment for those who have contraindications to the use of either pioglitazone or GLP-1 agonists.

Current standards of care in the management of Type 2 diabetes mellitus focus on the goal of cardiorenal risk reduction in high-risk patients on top of achievement and maintenance of glycemic and weight control^52^. Given the growing prevalence of NAFLD and its complications, it may be warranted to further discuss NAFLD in conversations highlighting the list of potential therapies beyond screening measures.

In the context of implications for future research, more randomized controlled trials are needed with (1) larger sample sizes to improve precision; (2) enrollment of a wider range of ethnic populations; (3) study objectives evaluating superiority or efficacy over placebo or standard of care (not only equivalence trials); and (4) use of intention-to-treat analysis in generating results to address bias due to deviations from intended interventions.

## Conclusion

The pooled meta-analysis suggests that sodium-glucose cotransporter 2 inhibitors slightly improve hepatic steatosis and fibrosis as compared to controls in adult patients with non-alcoholic fatty liver disease and Type 2 diabetes mellitus with low to moderate certainty of evidence.

### Supplementary Information

Supplementary Tables.

## Data Availability

The datasets used and/or analyzed during the current study are available from the corresponding author on reasonable request. The protocol was registered at the International Prospective Register of Systematic Reviews (PROSPERO) with the following ID number: CRD42022306396. All information provided in the protocol remained the same and no additional amendments were created.
