# Supplementary material for: Efficacy of sodium glucose cotransporter 2 inhibitors on hepatic fibrosis and steatosis in non-alcoholic fatty liver disease: an updated systematic review and meta-analysis
Source: Sci Rep. 2024 Jan 24;14:2122. doi: 10.1038/s41598-024-52603-5 (PMC10808406; doi:10.1038/s41598-024-52603-5)
Supplement: Supplementary file 1 — Supplementary Tables. [file 41598_2024_52603_MOESM1_ESM.pdf]

Efficacy of sodium glucose cotransporter 2 inhibitors on hepatic fibrosis and steatosis in non-alcoholic fatty liver disease: an updated systematic review and meta-analysis

Albert Macaire C. Ong Lopez[1], Janine Audrei T. Pajimna[2]

[1]*Department of Medicine, Section of Endocrinology, Diabetes and Metabolism, St. Luke's Medical Center- Quezon City*, 279 E Rodriguez Sr. Ave, Quezon City, Metro Manila, 1112, Philippines

[2]*Department of Medicine, St. Luke's Medical Center- Quezon City*, 279 E Rodriguez Sr. Ave, Quezon City, Metro Manila, 1112, Philippines

Supplementary material

Supplementary Table S1: PRISMA 2020 Checklist

Section and Topic	Item #	Checklist item	Location where item is reported (Page #)
TITLE			
Title	1	Identify the report as a systematic review.	1
ABSTRACT			
Abstract	2	See the PRISMA 2020 for Abstracts checklist.	1
INTRODUCTION			
Rationale	3	Describe the rationale for the review in the context of existing knowledge.	2
Objectives	4	Provide an explicit statement of the objective(s) or question(s) the review addresses.	2
METHODS			
Eligibility criteria	5	Specify the inclusion and exclusion criteria for the review and how studies were grouped for the syntheses.	3

Section and Topic	Item #	Checklist item	Location where item is reported (Page #)
Information sources	6	Specify all databases, registers, websites, organisations, reference lists and other sources searched or consulted to identify studies. Specify the date when each source was last searched or consulted.	4
Search strategy	7	Present the full search strategies for all databases, registers and websites, including any filters and limits used.	4
Selection process	8	Specify the methods used to decide whether a study met the inclusion criteria of the review, including how many reviewers screened each record and each report retrieved, whether they worked independently, and if applicable, details of automation tools used in the process.	4
Data collection process	9	Specify the methods used to collect data from reports, including how many reviewers collected data from each report, whether they worked independently, any processes for obtaining or confirming data from study investigators, and if applicable, details of automation tools used in the process.	4
Data items	10a	List and define all outcomes for which data were sought. Specify whether all results that were compatible with each outcome domain in each study were sought (e.g. for all measures, time points, analyses), and if not, the methods used to decide which results to collect.	4
	10b	List and define all other variables for which data were sought (e.g. participant and intervention characteristics, funding sources). Describe any assumptions made about any missing or unclear information.	4
Study risk of bias assessment	11	Specify the methods used to assess risk of bias in the included studies, including details of the tool(s) used, how many reviewers assessed each study and whether they worked independently, and if applicable, details of automation tools used in the process.	5
Effect measures	12	Specify for each outcome the effect measure(s) (e.g. risk ratio, mean difference) used in the synthesis or presentation of results.	5
Synthesis methods	13a	Describe the processes used to decide which studies were eligible for each synthesis (e.g. tabulating the study intervention characteristics and comparing against the planned groups for each synthesis (item #5)).	5
	13b	Describe any methods required to prepare the data for presentation or synthesis, such as handling of missing summary statistics, or data conversions.	5
	13c	Describe any methods used to tabulate or visually display results of individual studies and syntheses.	5
	13d	Describe any methods used to synthesize results and provide a rationale for the choice(s). If meta-analysis was performed, describe the model(s), method(s) to identify the presence and extent of statistical heterogeneity, and software package(s) used.	5

Section and Topic	Item #	Checklist item	Location where item is reported (Page #)
	13e	Describe any methods used to explore possible causes of heterogeneity among study results (e.g. subgroup analysis, meta-regression).	5
	13f	Describe any sensitivity analyses conducted to assess robustness of the synthesized results.	6
Reporting bias assessment	14	Describe any methods used to assess risk of bias due to missing results in a synthesis (arising from reporting biases).	6
Certainty assessment	15	Describe any methods used to assess certainty (or confidence) in the body of evidence for an outcome.	6
RESULTS			
Study selection	16a	Describe the results of the search and selection process, from the number of records identified in the search to the number of studies included in the review, ideally using a flow diagram.	6
	16b	Cite studies that might appear to meet the inclusion criteria, but which were excluded, and explain why they were excluded.	6
Study characteristics	17	Cite each included study and present its characteristics.	6
Risk of bias in studies	18	Present assessments of risk of bias for each included study.	7
Results of individual studies	19	For all outcomes, present, for each study: (a) summary statistics for each group (where appropriate) and (b) an effect estimate and its precision (e.g. confidence/credible interval), ideally using structured tables or plots.	7
Results of syntheses	20a	For each synthesis, briefly summarise the characteristics and risk of bias among contributing studies.	7-9
	20b	Present results of all statistical syntheses conducted. If meta-analysis was done, present for each the summary estimate and its precision (e.g. confidence/credible interval) and measures of statistical heterogeneity. If comparing groups, describe the direction of the effect.	7-9
	20c	Present results of all investigations of possible causes of heterogeneity among study results.	7-9
	20d	Present results of all sensitivity analyses conducted to assess the robustness of the synthesized results.	7-9
Reporting	21	Present assessments of risk of bias due to missing results (arising from reporting biases) for each synthesis assessed.	9

Section and Topic	Item #	Checklist item	Location where item is reported (Page #)
biases			
Certainty of evidence	22	Present assessments of certainty (or confidence) in the body of evidence for each outcome assessed.	9
DISCUSSION			
Discussion	23a	Provide a general interpretation of the results in the context of other evidence.	10
	23b	Discuss any limitations of the evidence included in the review.	11
	23c	Discuss any limitations of the review processes used.	11
	23d	Discuss implications of the results for practice, policy, and future research.	11
OTHER INFORMATION			
Registration and protocol	24a	Provide registration information for the review, including register name and registration number, or state that the review was not registered.	12
	24b	Indicate where the review protocol can be accessed, or state that a protocol was not prepared.	12
	24c	Describe and explain any amendments to information provided at registration or in the protocol.	12
Support	25	Describe sources of financial or non-financial support for the review, and the role of the funders or sponsors in the review.	12
Competing interests	26	Declare any competing interests of review authors.	12
Availability of data, code and other materials	27	Report which of the following are publicly available and where they can be found: template data collection forms; data extracted from included studies; data used for all analyses; analytic code; any other materials used in the review.	12

From: Page, M. J. *et al.* The Prisma 2020 statement: An updated guideline for reporting systematic reviews. *BMJ* (2021).

Supplementary Table S2. Baseline clinical characteristics of each study

Study author, year	Age [years] mean±SD		Female sex (%)		BMI (kg/m^2)		Hba1c (%)		FIB-4 index mean±SD	
	Cases	Controls	Cases	Controls	Cases	Controls	Cases	Controls	Cases	Controls
Chehrehgosha, 2021	50.5 ± 8.4	51.8 ± 7.8	42.9	37.8	30.9 ± 3.3	30.2 ± 4.4	8.08 ± 0.92	7.96 ± 0.62	0.94±0.42	0.94 ±0.43
Cho, 2021	63.5 ± 7.1	63.4 ± 10.2	44.4	50.0	75.1 kg ±15.8 (as weight)	74.6 ± 13.8 (as weight)	6.8 ± 0.6	6.9 ± 0.7	1.37 ± 0.59	1.32 ±0.50
Chu, 2022	46.79 ± 5.45	46.29 ± 5.42	55.5	60.0	28.54 ± 2.09	28.43 ± 2.13	8.75 ± 1.37	8.69 ± 1.34		
Elhini, 2022	47.75±8.59	47.36±8.55	66.25	68.75	32.67±4.34	32.02±5.68	8.97±1.39	7.98±1.18	1.03±0.66	0.80±0.29
Eriksson, 2018	65.0 ±6.5	65.6 ±6.1	23.81	19.04	30.5 ±2.8	30.3 ±3.1	7.44 ±0.80	7.38 ±0.56		
Han, 2020	56.7±11.8	56.7 ± 11.8	36.7	40.0	30.4 ± 5.4	30.2 ± 2.5	6.7 ± 0.7	6.6 ± 0.6		
Harrison, 2022	49.5±11.10	48.0 ±11.16	51.0	57	35.4 ±6.56	35.1 ±5.49	7.4 ±1.74	7.3 ±1.23	1.2 ±0.53	1.1 ±0.57
Hu, 2020	48.9±10.6	52.1±10.2	23.3	20.0	27.71±3.29	25.82±6.67	11.45±2.09	11.2±2.29		
Ito, 2017	57.3 ± 12.1	59.1 ± 9.8	56.0	47.0	30.7 ± 5.0	29.9 ± 6.2	8.5 ± 1.5	8.3 ± 1.4	1.44 ± 0.64	1.84 ± 1.13
Kinoshita, 2020	58.7 ± 1.6	58.0 ± 2.3	53.1	54.5	29.5 ± 0.8	28.4 ± 0.7	7.38 ± 0.16	7.57 ± 0.15		
Kuchay, 2018	49.1 ±10.3	50.7 ±12.8	41.1	40.0	30.0 ±3.8	29.4 ±3.1	9.0 ±1.0	9.1 ±1.4		

Shibuya, 2017	51 (47-62)	60 (53-66)	37.5	50.0	27.9 (26.2, 28.7)	27.2 (24.8, 32.1)	7.8 (7.2, 7.9)	7.4 (6.9, 7.7)		
Shimizu, 2018	56.2±11.5	57.1±13.8	42.42	37.50	27.6±4.7	28.3±3.5	8.37±1.48	7.70±1.24	1.50±0.93	1.11±0.64
Taheri, 2020	43.8 ±9.7	44.1 ±9.3	34.9	53.2	30.5 ±2.3	30.7 ±3.5	-	-	0.775 ±0.307	0.826 ±0.393
Takahashi, 2021	59.0 (46.8-64.3)	50.0 (48.0-68.8)	62.5	53.8	29.9 (27.2-32.3)	28.8 (25.7-32.9)	6.5 (6.1-7.1)	6.8 (6.3-7.0)		
Takeshita, 2022	59.0 (43.0–64.8)	50.5 (38.3–65.0)	65.0	30.0	31.0 ± 6.7	32.0 ± 8.8	7.9 (7.4–8.4)	8.2 (7.3–9.2)	1.10 (0.83–1.48)	0.95 (0.50–1.49)
Tobita, 2022	51.6 ± 12.6	41.8 ± 16.5	33.33	30.0	27.9 ± 4.3	28.7 ± 4.4	5.9 ± 0.4	5.8 ± 0.2	1.10 ± 0.36	1.16 ± 0.30
Yoneda, 2021	58.4±12.2	58.8±8.1	38.1	57.9	29.4±1.0	30.8±1.1	7.22±0.88	7.06±0.64	1.48±0.60	2.12±1.82

Supplementary Table S3. Baseline imaging biomarkers characteristics of each study

Study author, year	Controlled attenuation parameter (CAP) [dB/m] mean±SD		Liver-spleen (L/S) attenuation ratio mean±SD		MRI-PDFF [%] mean±SD		Liver stiffness measurement (LSM) [KPa] mean±SD	
	Cases	Controls	Cases	Controls	Cases	Controls	Cases	Controls
Chehrehgosha, 2021	317.37 ± 28.46	313.14 ± 30.40					6.83 ± 2.44	7.49 ± 2.65
Chu, 2022							9.87 ± 2.23	9.79 ± 2.26
Elhini, 2022					16.2 ±7.0	16.4 ±7.3		
Eriksson, 2018					17.3 ±9.1	15.1 ±6.5		
Han, 2020	305.5 ± 39.5	307.7 ± 37.0						
Hu, 2020	300.63±24.83	298±24.03					7.74±2.83	6.65±2.74
Harrison, 2022					21.1 ±8.99	23.3 ±9.42		
Ito, 2017			0.80 ±0.24	0.78 ± 0.26			0.80 ± 0.24	0.78 ± 0.26
Kinoshita, 2020			0.75 ± 0.04	0.73 ± 0.04				
Kuchay, 2018					16.2 ±7.0	16.4 ±7.3		
Shibuya, 2017			0.907 (0.637, 1.036)	0.991 (0.813, 1.118)				

Shimizu, 2018	314.1±61.0	306.0±34.3					9.49±6.05	8.01±5.78
Taheri, 2020	306.5 ±24.0	304.6 ±27.2					6.03 ±1.40	5.56 ±1.05
Takeshita, 2022	288.6 ±37.9	300.4 ±28.9					5.7 (4.3–7.3)	6.4 (4.7–11.3)
Yoneda, 2021					17.28±5.67	18.04±7.46		

Supplementary Table S4. Detailed inclusion and exclusion criteria of individual studies

Study author, year	Inclusion Criteria	Exclusion Criteria
Chehrehgosha, 2021	(1) Established Type 2 diabetes<br>(2) Diagnosed NAFLD with controlled attenuation parameter (CAP) C 238 dB/m in transient hepatic elastography	(1) Type 1 diabetes<br>(2) active or chronic hepatitis<br>cirrhosis and biliary disease<br>(3) heart failure defined as<br>NYHA class III and IV<br>(4) renal dysfunction<br>eGFR <45 ml/min/1.73 m^2)<br>(5) history of alcohol consumption [20 g per day in women and 30 g per day in men<br>(6) taking medications associated with fatty liver<br>NSAIDs, amiodarone, tamoxifen, sodium valproate, corticosteroids, methotrexate<br>(7) taking other fatty liver-related therapies such as vitamin E and trial medications (empagliflozin and pioglitazone)<br>(8) using supplements including<br>vitamin C, zinc, selenium, or antioxidant agents over the last months<br>(9) history of cardiovascular events within the past 3 months<br>(10) pregnancy and breastfeeding<br>(11) active cancer or history of cancer treatment over the past 2 years<br>(12) untreated thyroid disorder<br>(13) body mass index (BMI) ≥ 40 kg/m^2
Cho, 2021	(1) patients with Type 2 diabetes mellitus, aged 20–80 years, 6.5–8.5% of glycated hemoglobin (HbA1c)<br>(2) ≥23 kg/m2 of body mass index (BMI)<br>(3) estimated glomerular filtration rate ≥45 ml/min/1.73 m^2<br>(4) treatment with pioglitazone for >12 weeks	(1) Habitual drinkers<br>(2) FLI <30<br>(3) Current treatment with an SGLT2 inhibitor<br>(4) Hypersensitivity to dapagliflozin<br>(5) Severe or unstable retinopathy<br>(6) Severe liver damage (approximately Child-Pugh class C) or renal failure<br>(7) Severe diabetic ketosis, pre-coma, or coma

	(5) adequate diet and exercise	(8) Severe infection or trauma, or perioperative condition<br>(9) Pregnant or lactating<br>(10) Patients considered unsuitable for inclusion according to the physician's judgment.
Chu, 2022	(1) Type 2 diabetes mellitus with non-alcoholic fatty liver disease aged 18-70 years old, BMI 25-35 kg/m^2	(1) People with other types of diabetes<br>(2) People with acute complications<br>(3) Viral, drug-relateed, auto-immune and other acute or chronic liver diseases<br>(4) People with severe liver and kidney function damage<br>(5) People who have received treatments such as blood lipid regulation and blood sugar lowering within 1 month<br>(6) Those with contraindications to drug use<br>(7) those with malignant tumors, severe cardiovascular and cerebrovascular diseases, mental or intellectual disabilities who are unable to cooperate with the researcher
Elhini, 2022	(1) confirmed diagnosis of Type 2 diabetes using sulfonylurea (as Type 2 diabetes standard of care (SOC)) for at least the previous six months<br>(2) having any degree of liver steatosis on ultrasound	(1) Type 1 diabetes or ketoacidosis, (2) heavy alcohol consumers<br>(3) end-stage organ<br>Failure<br>(4) chronic renal failure (estimated eGFR below 60 ml/min/1.73 m^2, or on dialysis)<br>(5) other liver diseases (e.g., viral hepatitis, drug-induced liver disease, hepatocellular carcinoma, hepatobiliary disease, or autoimmune hepatitis)<br>(6) cardiac disease<br>(esp. NYHA classes III/IV)<br>(7) eating disorders or having previous bariatric surgery<br>(8) immunocompromised patients or with a history of inflammatory (acute or sclerosing<br>cholangitis), immunological, or malignant diseases<br>(9) pregnant or lactating females<br>(10) contraindication or hypersensitivity to drugs or MRI procedures

Eriksson, 2018	(1) Type 2 diabetes, aged 40–75 years treated with a stable dose of metformin or sulfonylurea alone or in combination for at least 3 months,<br>(2) MRI-PDFF >5.5%<br>(3) BMI of 25–40 kg/m^2	(1) use of SGLT2 inhibitors,<br>n-3 fatty acids, insulin or glucagon-like peptide 1 receptor agonists<br>(2) a history of hepatic disease, (3) creatinine clearance <60 ml/min (Cockcroft–Gault), (4) inability to undergo MRI scanning<br>(5) significant alcohol intake (>14 drinks per week)
Han, 2020	(1) Type 2 diabetes receiving metformin and pioglitazone combination therapy for at least 8 weeks and naive to treatment with any SGLT2 inhibitors<br>(2) NAFLD, as diagnosed by abdominal ultrasound and<br>confirmed by radiologic specialists.	(1) diagnosis of type<br>1 diabetes, gestational diabetes, or any diabetes diagnosis other than type 2 diabetes<br>(2) history of addiction to alcohol, heavy alcohol consumption (>210 g/week for men or > 140 g/week for women)<br>(3) aspartate aminotransferase (AST), alanine aminotransferase (ALT), or bilirubin levels<br>more than three times the upper normal limit<br>(4) other causes of liver disease (e.g., active viral or autoimmune hepatitis), liver cirrhosis, or hepatocellular carcinoma<br>(5) estimated glomerular filtration<br>rate (eGFR) < 60 ml/min/1.73 m^2; (6) medication associated with fatty liver disease (e.g., amiodarone,<br>methotrexate, tamoxifen, or valproate) or weight loss<br>(7) pregnant or nursing women
Harrison, 2022	(1) diagnosis of T2DM based on HbA1c levels ranging between 6.5% and 10%.<br>(2) presence of NASH based on liver biopsy done within 2 years of randomization, with fibrosis levels of F1, F2 or F3 in the absence of a histological diagnosis of alternative chronic liver disease, and ALT ≥ 50 U l−1 (males) or ≥35 U l−1 (females) at screening or phenotypic diagnosis of NASH based on	(1) history or presence of concomitant liver diseases and cirrhosis, hepatic decompensation, or severe liver impairment<br>(2) type 1 diabetes and uncontrolled diabetes (Hba1c ≥ within 60 days before enrollment)<br>(3) using GLP-1 agonist, SGLT-2 inhibitors, thiazolidinediones, and FXR agonists, and other weight loss medications<br>(4) eGFR ≤ 45 ml/min/1.73 m^2)<br>(5) donated or loss of more than 400 ml of blood within 8 weeks<br>(6) contraindications to MRI

	presence of ALT ≥ 50 U l−1 (males) or ≥35 U l−1 (females)<br>(3) BMI ≥ 23 kg/m^2in Asian-heritage patients or ≥27 kg/m^2 in patients other than Asian race	(7) Platelet count < 120 x 10^9/L<br>(8) HIV<br>(9) pregnant, lactating women or women of child bearing potential<br>(10) Use of CYP3A4/5 inhibitors<br>(11) patients with ketoacidosis, lactic acidosis, or hyperosmolar Coma<br>(12) planned or prior bariatric surgery<br>(13) Patients on treatment with the following medicines unless they are on a constant dose for ≥3 months before randomization: anti-diabetic medications, insulin (if ≥25% change in dose), beta-blockers, thiazide diuretics, fibrates, statins, niacin, ezetimibe, vitamin E (if doses > 400 IU/day; doses > 800<br>IU/day are prohibited), thyroid hormone, psychotropic medications, estrogen<br>or estrogen containing birth control<br>(14) Symptomatic genital or urinary tract infection within 4 weeks before first<br>study visit<br>(15) untreated malignancy<br>(16) drug or alcohol abuse<br>(17) history of non-adherence to medical regimens
Hu, 2022	(1) All met the 1999 World Health Organization (WHO) diagnostic criteria for type 2 diabetes; Diabetes duration ≤2 y; Age 20-65 y<br>(2) Color Doppler ultrasound diagnosis of fatty liver	(1) Type 1 diabetes<br>(2) Diabetes acute and chronic complications<br>(3) Severe infection<br>(4) Severe liver and kidney insufficiency<br>(5) cardiac insufficiency<br>(6) Long term heavy drinkers (drinking equivalent The amount of ethanol is ≥140 g/w for men and ≥70 g/w for women);<br>(7) have autoimmune liver disease, viral hepatitis, drug- induced liver disease, and cirrhosis
Ito, 2017	(1) Type 2 diabetes receiving diet and exercise therapy alone or with oral	(1) eGFR of ≤ 45 ml/min/1.73 m^2, serum creatinine > 1.5 mg/dL<br>(2) history of serious diabetes complications, findings suggestive of

	hypoglycemic agents other than SGLT2 inhibitors and<br>thiazolidinediones and/or insulin.<br>(2) NAFLD, findings suggesting hepatic steatosis and<br>hepatic dysfunction on clinical laboratory tests or on imaging studies (e.g., computed tomography [CT] or ultrasound)<br>(3) alcohol consumption volume <30 g/day (men) or <20 g/day (women),	insulin dependency,<br>(3) heart failure (NYHA Class III or IV), (4) history of myocardial or cerebral<br>Infarction<br>(5) findings suggestive<br>of decompensated cirrhosis, and other causes of liver disease (e.g., viral or autoimmune hepatitis)
Kinoshita, 2020	(1) age ≥20 years<br>(2) hemoglobin A1c (HbA1c) ≥6.5%<br>(3) body mass index (BMI) ≥22 kg/m^2<br>(4) alanine aminotransferase<br>(ALT) ≥25 units/L (men) or ≥17 units/L (women) at screening,<br>(5) stable dose of diabetes medicine for ≥1.5 month<br>(6) L/S ratio < 1.0	(1) alcohol use (>30 g/day for men, >20 g/day for<br>women)<br>(2) previous treatment during the past 3 months (with<br>insulin, SGLT2 inhibitor, thiazolidinediones or sulfonylurea)<br>(3) diabetic coma<br>(4) renal dysfunction (estimate glomerular filtration rate<br><45 mL/min)<br>(5) cardiac failure<br>(6) liver diseases (viral hepatitis, alcoholic hepatitis, autoimmune liver disease or liver cirrhosis), (7) use of steroid and/or<br>immunosuppressant<br>(8) pregnant, possible pregnancy<br>and/or breast-feeding<br>(9) when the researcher deemed an individual<br>inappropriate as a study participant<br>(10) individuals who did not visit the hospital for ≥1 month and/or who had <70% medication compliance.
Kuchay, 2018	(1) > 20 years of age<br>(2) uncontrolled type 2 diabetes (HbA1c > 7.0% to <10.0%)	(1) highly uncontrolled diabetes<br>(HbA1c > 10.0%)<br>(2) alcohol intake > 30 g/day (three drinks per day) within the

	(3) documented hepatic steatosis (MRI-PDFF > 6%)	previous 10 years or > 10 g/day within the previous year<br>(3) evidence of other forms of liver disease, including hepatitis B and C infection, autoimmune hepatitis, drug-induced liver disease on the basis of exposure and history, and biliary duct obstruction<br>(4) history of gastrointestinal bypass (5) use of drugs known to cause hepatic steatosis (e.g., amiodarone, valproate, tamoxifen, methotrexate, steroids)<br>(6) recent initiation or change of antidiabetic drugs that influence liver fat, including thiazolidinediones and GLP-1 agonists, or recent initiation of any SGLT-2 inhibitor, within 90 days of randomization<br>(7) evidence of cirrhosis or hepatocellular carcinoma<br>(8) positive HIV test<br>(9) active substance abuse;<br>(10) pregnant or trying to become pregnant<br>(11) renal insufficiency (eGFR <90 ml/min/1.73 m^2<br>(12) contraindications to empagliflozin use<br>(13) contraindications to MRI
Shibuya, 2017	(1) HbA1c concentration, 6.0%–10.0%<br>(2) age, 20–70 years<br>(3) absence of hepatitis B and hepatitis C<br>(4) alcohol intake not exceeding 140 g/week in women and 210 g/week in men<br>(5) fatty liver on basis of CT scan or abdominal sonography	(1) patients with >5% change in body weight within 3 months<br>(2) histories of myocardial infarction, angina pectoris, or cerebral apoplexy and those taking prescribed diuretics<br>(3) having severe renal dysfunction (eGFR < 45 ml/min/1.73 m^2)<br>(4) unsuitable for participation for other medical reasons

Shimizu, 2018	(1) type 2 diabetes combined with NAFLD on the basis of liver dysfunction (persistent elevation of ALT ≥ the upper limit for our laboratory), the presence of fatty liver on ultrasonography, low daily alcohol intake (less than 30 g for men and less than 20 g for women), and exclusion of other liver diseases such as chronic hepatitis B and C, autoimmune hepatitis)<br>(2) at least 20 years old<br>(3) HbA1c level of 6.0–12.0% on stable therapy with one to three oral antidiabetic agents with or without insulin for at least 3 months.	(1) other liver diseases such as chronic hepatitis B and C, autoimmune hepatitis<br>(2) alcohol intake > 30 g/day for men and > 20 g/day for women
Taheri, 2020	(1) individuals aged 20–65 years,<br>(2) ALT > 45 mg/dL and > 30 mg/dL respectively in men and women<br>(3) one of these items (fatty liver grade ≥ 2 in liver ultrasonography or controlled attenuation parameter (CAP) in fibroscan > 230 decibels per meter	(1) FPG ≥126 mg/dL (7.0 mmol/L) or a HbA1c level ≥ 6.5%<br>(2) alcohol consumption greater than 20 g per day in women or greater than 30 g in men for at least three consecutive months over the past 5 years<br>(3) history of acute or chronic liver, biliary, or cirrhotic diseases; (4) heart failure (NYHA class 2–4)<br>(5) renal failure (eGFR < 45 ml/min/1.73 m^2)<br>(6) taking medications associated with fatty liver such as NSAIDs, amiodarone, tamoxifen, sodium valproate, corticosteroids, Methotrexate<br>(7) using supplements including vitamin E, vitamin C, zinc, and selenium or antioxidant agents over the last 3 months<br>(8) history of cardiovascular events within the past 3 months

		(9) pregnancy or breastfeeding; (10) active cancer or history of cancer treatment over the past 2 years<br>(11) untreated thyroid disorder;<br>(12) BMI > 40 kg/m^2
Takahashi, 2021	(1) age 20-80 years<br>(2) histological diagnosis of NAFLD by the nominated study pathologists<br>(3) HbA1c ≥ 6.0%<br>(4) no existing medication with an SGLT2 inhibitor, pioglitazone, GLP-1 agonist, or insulin<br>(5) steatosis involving ≥5% of the hepatic parenchyma of a liver biopsy performed at baseline.	(1) presence of a severe complication of diabetes, including severe diabetic nephropathy (eGFR <30 mL/min/1.73 m^2) and/or diabetic retinopathy at a more severe stage than simple diabetic retinopathy<br>(2) diagnosis of type 1 diabetes<br>(3) history of severe CVD, including ischemic heart disease, chronic heart failure, cerebral infarction, and/ or peripheral vascular disorders<br>(4) presence of etiological factors suggesting a diagnosis of a different liver disease, including habitual alcoholic intake (ethanol consumption >30 g/day and >210 g/week in men and >20 g/day and >140 g/week in women)<br>(5) positivity for hepatitis B surface antigen, positivity for hepatitis C antibody, and abnormal serum thyroid hormone concentration; and<br>(6) diagnosis of autoimmune liver disease, drug-induced hepatotoxicity, hemochromatosis, or Wilson disease
Takeshita, 2022	(1) A diagnosis of "definite" NAFLD on liver biopsy obtained within 3 months of screening.<br>(2) ≥20 years of age at the time of the initial screening<br>(3) Patients with type 2 diabetes mellitus at the time of screening need to have glycemic	(1) hepatic virus infections (hepatitis B and C, cytomegalovirus, and Epstein–Barr virus), autoimmune hepatitis, primary biliary cirrhosis, sclerosing cholangitis, hemochromatosis, alpha-1-antitrypsin deficiency, Wilson’s disease<br>(2) history of parenteral nutrition, (3) use of agents known to induce steatosis (e.g., valproate, amiodarone, or vitamin E)<br>(4) hepatic injury caused by substance abuse

	control (HbA1c of ≥7%) and have been managed by either diet and/or a stable dose of hypoglycemic agents for at least 4 weeks.	(5) current consumption of more than 20 g of alcohol daily<br>(6) No clinical evidence of hepatic decompensation<br>(7) tofogliflozin or glimepiride hypersensitivity or contraindications<br>(8) history of type 1 diabetes<br>(9) history of ketoacidosis<br>(10) history of symptoms of hypoglycemia<br>(11) treatment with SGLT2 inhibitor including tofogliflozin within 4 weeks of screening<br>(12) glinide and sulfonylurea use within 4 weeks of screening<br>(13) concomitant corticosteroid therapy<br>(14) poorly controlled unstable diabetes (ketoacidosis or an increase in HbA1c of more than 3% in the 12 weeks before screening), (15) poorly controlled hypertension or systolic blood pressure greater than 160 mmHg or diastolic blood pressure greater than 100 mmHg, (16) artificial dialysis or moderate renal dysfunction<br>(17) poorly controlled dyslipidemia, (18) presence of a severe health problem, not being suitable for the study<br>(19) pregnant or breastfeeding<br>(20) inability to participate in the study (including psychiatric and psychosocial problems)
Tobita, 2022	(1) presence of hepatorenal contrast and increased hepatic echogenicity in abdominal ultrasonography findings obtained using a 3.5-MHz transducer (ARIETTA S70; Hitachi, Ltd., Tokyo, Japan)<br>(2) daily alcohol consumption lower than 20 g/day<br>(3) negative for Wilson's disease, hemochromatosis, and drug-induced liver injury, as well as the	(1) the presence of severe heart, liver, or kidney disease

	presence of<br>hepatitis B surface antigen, and anti-HCV, antinuclear, and<br>antimitochondrial antibodies<br>(4) Age ≥ 20 years old<br>(5) No Type 2 diabetes<br>(6) ALT ≥ 31 and < 200 IU/L	
Yoneda, 2021	(1) Both men and women aged 20–74 years<br>(2) Type 2 diabetes patients who had undergone diet and exercise therapies<br>(3) Patients with HbA1c ≥6.5% (48 mmol/mol) within 90 days of primary registration<br>(5) Patients diagnosed with non-alcoholic fatty liver disease<br>(6) Patients with ALT levels beyond the institutional standard levels (42 IU/L for men and 23 IU/L for women) within 90 days of primary registration	(1) Patients with drinking habits (men, more than 30 g/day; women, more than 20 g/day<br>converted to ethanol)<br>(2) Patients diagnosed with viral hepatitis (including patients under treatment and carriers)<br>(3) Patients with other types of hepatitis or liver disorder (e.g., drug-induced, autoimmune)<br>(4) Patients diagnosed with liver cirrhosis<br>(5) Patients with serious liver dysfunction (Child-Pugh B or C)<br>(6) Patients with PLT <150×103/ µL within 90 days of primary registration<br>(7) Patients with BMI <22 kg/m^2 within 90 days of primary registration<br>(8) Patients with ALT >5 times the upper limit of the standard value within 90 days of primary<br>registration<br>(9) Patients with serious renal dysfunction or with eGFR <60 $mL/min/1.73\ m^2$ within 90 days<br>of primary registration<br>(10) Patients with type 1 diabetes mellitus or with HbA1c ≥9.0% (75 mmol/mol) within 90 days<br>of primary registration<br>(11) Patients using SGLT2 inhibitor, pioglitazone, insulin, or GLP-1

		receptor agonists<br>(12) Patients taking vitamin E<br>(13) Patients with any contraindications to MRI (e.g., pacemaker)<br>(14) Patients unable to undergo MRI (e.g., cannot hold breath, patients with excessive iron<br>deposition)<br>(15) Patients with heart failure of more than NYHA III at present or in the past
